# Microbial dysbiosis and mortality during mechanical ventilation: a prospective observational study

**DOI:** 10.1186/s12931-018-0950-5

**Published:** 2018-12-07

**Authors:** Daphnée Lamarche, Jennie Johnstone, Nicole Zytaruk, France Clarke, Lori Hand, Dessi Loukov, Jake C. Szamosi, Laura Rossi, Louis P. Schenck, Chris P. Verschoor, Ellen McDonald, Maureen O. Meade, John C. Marshall, Dawn M. E. Bowdish, Tim Karachi, Diane Heels-Ansdell, Deborah J. Cook, Michael G. Surette, for the PROSPECT Investigators, Canadian Critical Care Trials Group, Canadian Critical Care Translational Biology Group

**Affiliations:** 1 Department of Biochemistry and Biomedical Sciences, McMaster University, Hamilton, ON Canada; 2 Institute for Infectious Disease Research, McMaster University, Hamilton, ON Canada; 3 Farncombe Family Digestive Health Research Institute, McMaster University, Hamilton, Ontario Canada; 4 Department of Medicine, University of Toronto, Toronto, ON Canada; 5 Dalla Lana School of Public Health, University of Toronto, Toronto, ON Canada; 6 Public Health Ontario, Toronto, ON Canada; 7 St. Joseph’s Healthcare, Hamilton, ON Canada; 8 Department of Health Research Methods, Evidence and Impact, McMaster University, Hamilton, ON Canada; 9 Hamilton Health Sciences, Hamilton, ON Canada; 10 Department of Pathology and Molecular Medicine, Hamilton, ON Canada; 11 Department of Medicine, McMaster University, Health Sciences Bldg, 3N8F, 1280 Main Street West, Hamilton, ON L8S 4K1 Canada; 12 Department of Surgery, University of Toronto, Toronto, ON Canada; 13 Interdepartmental Division of Critical Care, University of Toronto, Toronto, ON Canada

**Keywords:** Microbiome, Critical illness, Microbial diversity, Respiratory tract microbiota, Gastrointestinal tract microbiota

## Abstract

**Background:**

Host-associated microbial communities have important roles in tissue homeostasis and overall health. Severe perturbations can occur within these microbial communities during critical illness due to underlying diseases and clinical interventions, potentially influencing patient outcomes. We sought to profile the microbial composition of critically ill mechanically ventilated patients, and to determine whether microbial diversity is associated with illness severity and mortality.

**Methods:**

We conducted a prospective, observational study of mechanically ventilated critically ill patients with a high incidence of pneumonia in 2 intensive care units (ICUs) in Hamilton, Canada, nested within a randomized trial for the prevention of healthcare-associated infections. The microbial profiles of specimens from 3 anatomical sites (respiratory, and upper and lower gastrointestinal tracts) were characterized using 16S ribosomal RNA gene sequencing.

**Results:**

We collected 65 specimens from 34 ICU patients enrolled in the trial (29 endotracheal aspirates, 26 gastric aspirates and 10 stool specimens). Specimens were collected at a median time of 3 days (lower respiratory tract and gastric aspirates; interquartile range [IQR] 2–4) and 6 days (stool; IQR 4.25–6.75) following ICU admission. We observed a loss of biogeographical distinction between the lower respiratory tract and gastrointestinal tract microbiota during critical illness. Moreover, microbial diversity in the respiratory tract was inversely correlated with APACHE II score (*r* = − 0.46, *p* = 0.013) and was associated with hospital mortality (Median Shannon index: Discharged alive; 1.964 vs. Deceased; 1.348, *p* = 0.045*)*.

**Conclusions:**

The composition of the host-associated microbial communities is severely perturbed during critical illness. Reduced microbial diversity reflects high illness severity and is associated with mortality. Microbial diversity may be a biomarker of prognostic value in mechanically ventilated patients.

**Trial registration:**

ClinicalTrials.gov ID NCT01782755. Registered February 4 2013.

**Electronic supplementary material:**

The online version of this article (10.1186/s12931-018-0950-5) contains supplementary material, which is available to authorized users.

## Background

The human body harbours trillions of bacterial cells on and within its surfaces and mucous membranes [1]. These microorganisms (microbiota) are largely commensals and mutualists that can confer health advantages to the host. The microbiome is essential for numerous features of host physiology, including metabolism (by degrading otherwise non-digestible molecules), resistance to infection (e.g., via colonization resistance), and immune maturation and homeostasis [2, 3]. Normally, the microbiota of healthy individuals is stable over time, although it is sensitive to changes in lifestyle, diet, and illnesses [1, 4]. Perturbations of these microbial ecosystems can be associated with several diseases, including inflammatory bowel disease, and *Clostridium difficile* infection, as well as conditions associated with critical illness (i.e., sepsis, acute respiratory distress syndrome, and multiple organ dysfunction syndrome) [1, 5–9].

The microbiota of patients in the intensive care unit (ICU) fluctuates considerably due to acute disease states associated with critical illness, and common interventions such as mechanical ventilation, antimicrobials, gastric acid suppression, and enteral nutrition [6, 10]. Studies using culture-dependent and culture-independent methods have demonstrated that microbial diversity in the gastrointestinal (GI) and respiratory tracts of critically ill patients decreases following ICU admission, and that critically ill patients experience pronounced disturbances of their microbial communities which become more severe over time [8, 11–17].

The consequences of microbial dysbiosis on illness severity and mortality have been relatively unexplored, particularly in the lower respiratory tract. A better understanding of microbial disturbances in the ICU setting and their impact on clinical outcomes is needed, given the emerging evidence suggesting that therapeutics targeting the microbiota in critical illness may be promising to prevent or treat complications [18–21].

The objectives of this prospective observational study were to investigate the microbial composition at distinct anatomical sites of the respiratory and GI tracts during critical illness, and to evaluate whether the microbial diversity in the first week of the ICU stay of mechanically ventilated patients is associated with illness severity and mortality.

## Methods

### Subject recruitment

We recruited critically ill patients 18 years of age or older receiving invasive mechanical ventilation from a medical-surgical and a neuro-trauma ICU in 2 hospitals in Hamilton, Canada. Samples were collected between October 2013 and June 2014 as a translational study nested within a multicenter pilot randomized blinded trial (PROSPECT, NCT02462590) testing the effect of the probiotic *Lactobacillus rhamnosus* GG versus placebo on the risk of ventilator-associated pneumonia (VAP) and other infections [22]. Further information concerning patient recruitment is available in the Additional file 1.

Healthy donors included in this study were individuals older than 18 years of age without comorbidities who had not received antibiotics in the 6 months before sample collection. Healthy donors were included to investigate the microbial compositional differences between healthy and critically ill individuals; they also participated in other ongoing studies in our laboratory [23–25].

This study was approved by the Hamilton Integrated Research Ethic Board. All participants or their substitute decision makers provided written informed consent prior to participation.

### Sample collection

Endotracheal tube aspirate (ETA), gastric tube aspirate (GA), fecal samples and peripheral venous blood were aseptically collected and transferred by the research coordinator at each ICU. To limit confounders due to probiotic administration, the first available sample following study enrollment from each patient at each body site was used to compare differences in the composition of the microbiota between anatomical sites. All samples included were collected before the 7th day in the ICU. For the healthy cohort, nasopharyngeal and oropharyngeal swabs, fecal samples and bronchoalveolar lavages (BAL) were collected as described elsewhere [23, 24, 26].

### DNA extraction, 16S rRNA gene sequencing, and sequence processing

The genomic DNA extraction and amplification for sequencing on the Illumina MiSeq platform by the McMaster Genomics Facility (Hamilton, Canada) was performed as described previously [27]. Paired-end sequences of the v3 region of the 16S rRNA gene were processed through a standardized workflow [28]. A negative genomic extraction and sequencing controls were conducted to ensure that sequencing contamination was not an issue for low-biomass samples. Our sequencing data and metadata is available at NCBI SRA BioProject PRJNA428805 (SRA accession: SRP128586). Supplementary information is available in the Additional file 1.

### Data analysis

The α and β-diversity estimates were generated in R (R Core Team 2016) [29] using the ‘phyloseq’ package [30]. β-diversity was calculated on the proportionally normalized operational taxonomic unit (OTU; i.e., a proxy for bacterial ‘species’) table excluding singletons, and OTUs classified as non-bacterial. The α-diversity was calculated using the OTU table excluding only non-bacterial OTUs and rarefied to 2800 reads per sample 100 times, and the mean value of the α-diversity measurements was used. Three α-diversity metrics were included in this investigation; the Shannon and Simpson diversity indices account for richness (i.e., number of taxa) and evenness (i.e., taxa relative abundances), while the metric Observed Species accounts only for richness [31]. The within-body site distance-to-centroid was calculated on a Bray-Curtis distance matrix using the function betadisper from the R package ‘Vegan’ [32]. The UPGMA consensus tree was generated in QIIME with support established using jackknife [33]. The correlation analysis between patients’ metadata and α-diversity measurements was performed using the R packages ‘Hmisc’ and ‘corrplot’, using the Spearman’s rank correlation coefficient [34, 35]. The Kaplan-Meier estimate was generated using the R packages ‘survival’ and ‘survminer’ [36, 37]. Survival analysis using Simpson diversity was not included since the same sets of patients were generated in both groups resulting in identical results than using Shannon diversity. The impact of concomitant antimicrobial exposure with sample collection on ETA α-diversity was investigated. Additional investigation on the influence of individual antimicrobials was unsuitable given our limited number of samples.

### Statistical analysis

Differences across body sites in community composition (biogeography) was investigated using a permutational multivariate analysis of variance (PERMANOVA), performed with the ‘Vegan’ package in R [32] on a Bray-Curtis distance matrix, using 100,000 permutations. Differences in within-site inter-sample Bray-Curtis and centroid distances were tested with the ‘lmerTest’ package in R [38], using a linear mixed model with fixed effects of cohort (i.e., Healthy and ICU) and anatomical site, and a random effect of patient on intercept. Compositional differences between the ICU patients and healthy donors were tested in QIIME using a Kruskal-Wallis test. *P* values were adjusted using Benjamini-Hochberg false discovery rate (FDR) correction. The α-diversity, bacterial load, serum cytokines and clinical markers results are presented using the median and interquartile range and were analyzed with a Mann-Whitney test using GraphPad Prism version 6.0 (La Jolla, CA, USA). Correlation analysis was followed with FDR correction to account for multiple-testing [29]. For the survival analysis, differences in mortality between groups were tested using a log-rank test [29]. The two population proportions (deceased vs. discharged alive within the low and high diversity groups) were compared using a Fisher’s exact test using GraphPad Prism. The significance threshold was set at *p* < 0.05.

### Sensitivity analysis

To evaluate whether study product exposure influenced the outcome, *L. rhamnosus* GG sequences (OTU5) were removed from the sensitivity analysis. Results of these analyses were then compared to ensure that the impact of the ingested probiotic administration was minimal and that the study results were not influenced by the presence of *L. rhamnosus* GG. More information is available in the Additional file 1.

## Results

### Demographics of the critically ill cohorts and healthy controls

In total, 34 mechanically ventilated critically ill patients were enrolled; demographic data are presented in Table 1. Patients were a mean (standard deviation [SD]) of 66.6 (10.9) years of age, had an Acute Physiology and Chronic Health Evaluation (APACHE) II score of 25.5 (8.5), and 14/34 (41.2%) were female (Table 1). Patients were admitted to the ICU for medical conditions (*n* = 31, 91.2%), surgical conditions (*n* = 1, 2.9%), or trauma (*n* = 2, 5.9%). The median ICU length of stay was 11.5 days (IQR 7–20.5) and 22 (64.7%) patients were alive at hospital discharge.

**Table 1:** Demographics and characteristics of ICU patients

Characteristics	Patients (*n* = 34)
Age (years), mean (SD)	66.6 (10.9)
APACHE II, mean (SD)	25.5 (8.5)
Female, n (%)	14 (41.2)
Type of patient, *n* (%)	
Medical	31 (91.2)
Surgical	1 (2.9)
Trauma	2 (5.9)
Admitting Diagnosis, *n* (%)	
Pneumonia	13 (38.2)
Sepsis	8 (23.5)
Chronic obstructive pulmonary disease exacerbation	2 (5.9)
Congestive heart failure	2 (5.9)
Respiratory arrest	2 (5.9)
Trauma	2 (5.9)
Alcohol withdrawal	1 (2.9)
Cardiac arrest	1 (2.9)
Cardiogenic shock	1 (2.9)
Laminectomy	1 (2.9)
Renal failure	1 (2.9)
ICU length of stay (days), median (quartile 1, quartile 3)	11.5 (7, 20.5)
Hospital length of stay (days), median (quartile 1, quartile 3)	32 (15, 55)
ICU Mortality, *n* (%)	5 (14.7)
Hospital Mortality, *n* (%)	12 (35.3)

In total, 65 samples were collected including 29 ETAs, 26 GAs and 10 stool specimens (Additional file 1: Table S1). Data collection was a median of 3 days following ICU admission for ETA and GA samples (IQR 2–4), and 6 days for stool samples (IQR 4.3–6.8).

Samples from 35 healthy adults were included to establish reference points for the biogeography of the healthy microbiome for comparison to the ICU cohort. One specimen type was collected per healthy donor with the exception of the nasopharynx (NP) and oropharynx (OP) swabs, which were both collected from the same individual. In total, 42 samples from 35 healthy adults consisting of 7 BALs, 7 NP swabs and 7 OP swabs, and 21 stool specimens were included (Additional file 1: Table S2).

### Loss of biogeography within the ICU cohort compared to healthy controls

**Fig. 1:**
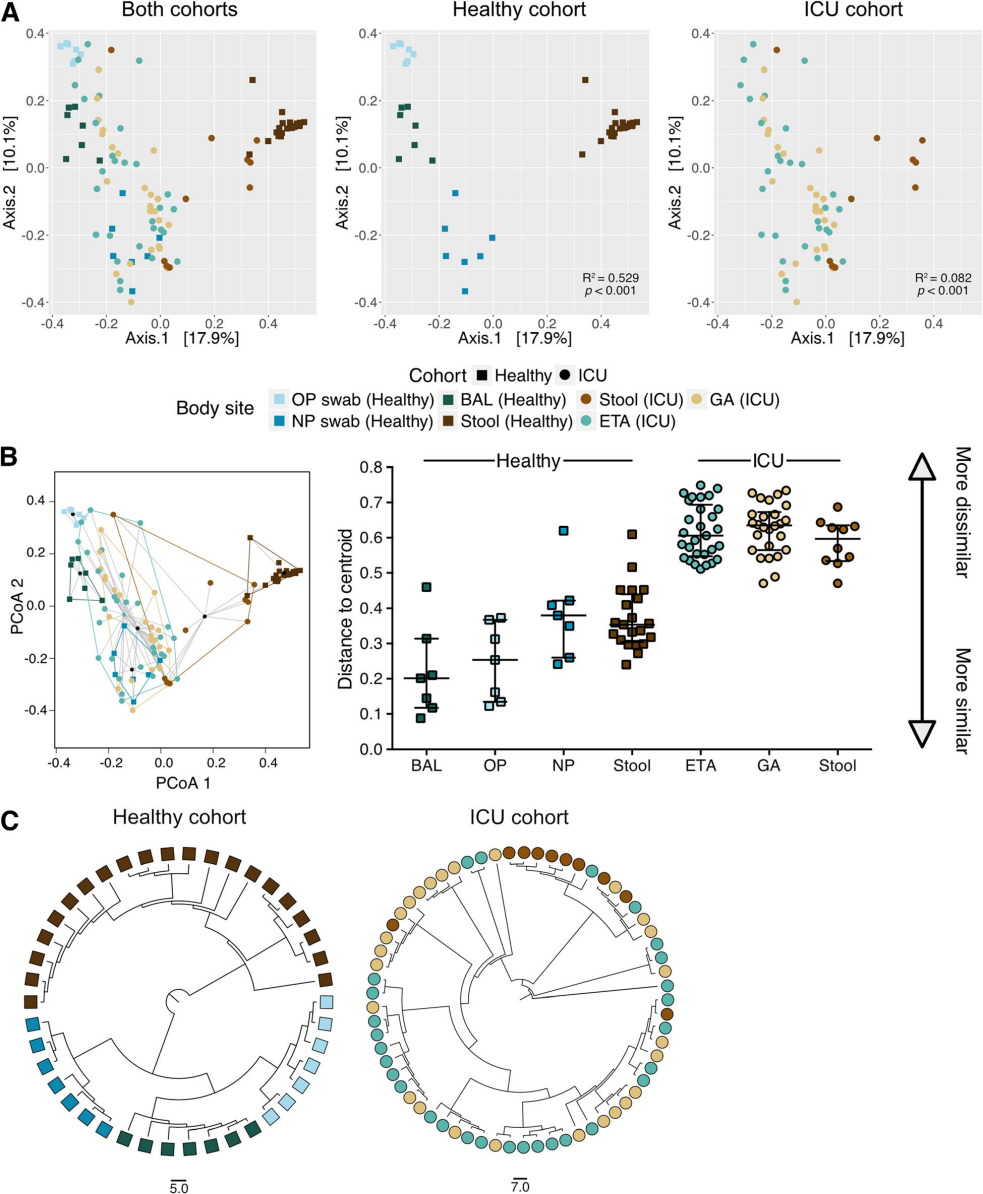
Lack of microbial consensus and loss of biogeographical distinction in ICU patients. Principal coordinate analysis (PCoA) ordination using the Bray-Curtis dissimilarity metric between the ICU and healthy cohorts demonstrate that samples collected from the healthy cohort tend to cluster per body sites (PERMANOVA, *p* < 0.001, R^2^ = 0.529) whereas the samples from different anatomical sites tend to overlap in the ICU cohort (PERMANOVA, *p* < 0.001, R^2^ = 0.082; **a**). The ordination plot of group dispersions within body site demonstrates a lack of compositional homogeneity within anatomical sites in the ICU cohort (*p* < 0.001; **b**). The overlaying lines on the scatter plot show the median distances between the cluster’s centroid displayed with a black circle and each samples within the group and the interquartile range of each site. UPGMA dendogram showing the Bray-Curtis dissimilarity between specimens displays a perfect segregation of samples in the healthy cohort based on collections sites. This is not observed in the ICU cohort (**c**)

We compared the microbial community structures of patients admitted to the ICU to a healthy cohort to investigate the homogeneity of microbial composition within each body site and to observe any change in biogeography during critical illness (Fig. 1). The β-diversity was measured using the Bray-Curtis dissimilarity metric. Microbial communities segregate substantially and significantly by body site in healthy individuals (PERMANOVA, *p* < 0.001, R^2^ = 0.529; Fig. 1a). However, differences in composition by anatomical site were considerably less pronounced in the ICU cohort than healthy controls (PERMANOVA, *p* < 0.001, R^2^ = 0.082; Fig. 1a). Indeed, there is minimal visible clustering in ICU specimen types (Fig. 1b), and the distance-to-centroid within-body site is significantly higher in the ICU cohort than the healthy controls (*p* < 0.001, Fig. 1b). Comparison of the pairwise Bray-Curtis dissimilarity among individual body site specimens from the ICU and the healthy cohort demonstrates significantly higher heterogeneity within the ICU cohort for samples collected at those body sites (*p* < 0.0001; Additional file 1: Figure S1). These results show that the distinct biogeographical composition of the microbiome at different body sites is attenuated in ICU patients.

**Fig. 2:**
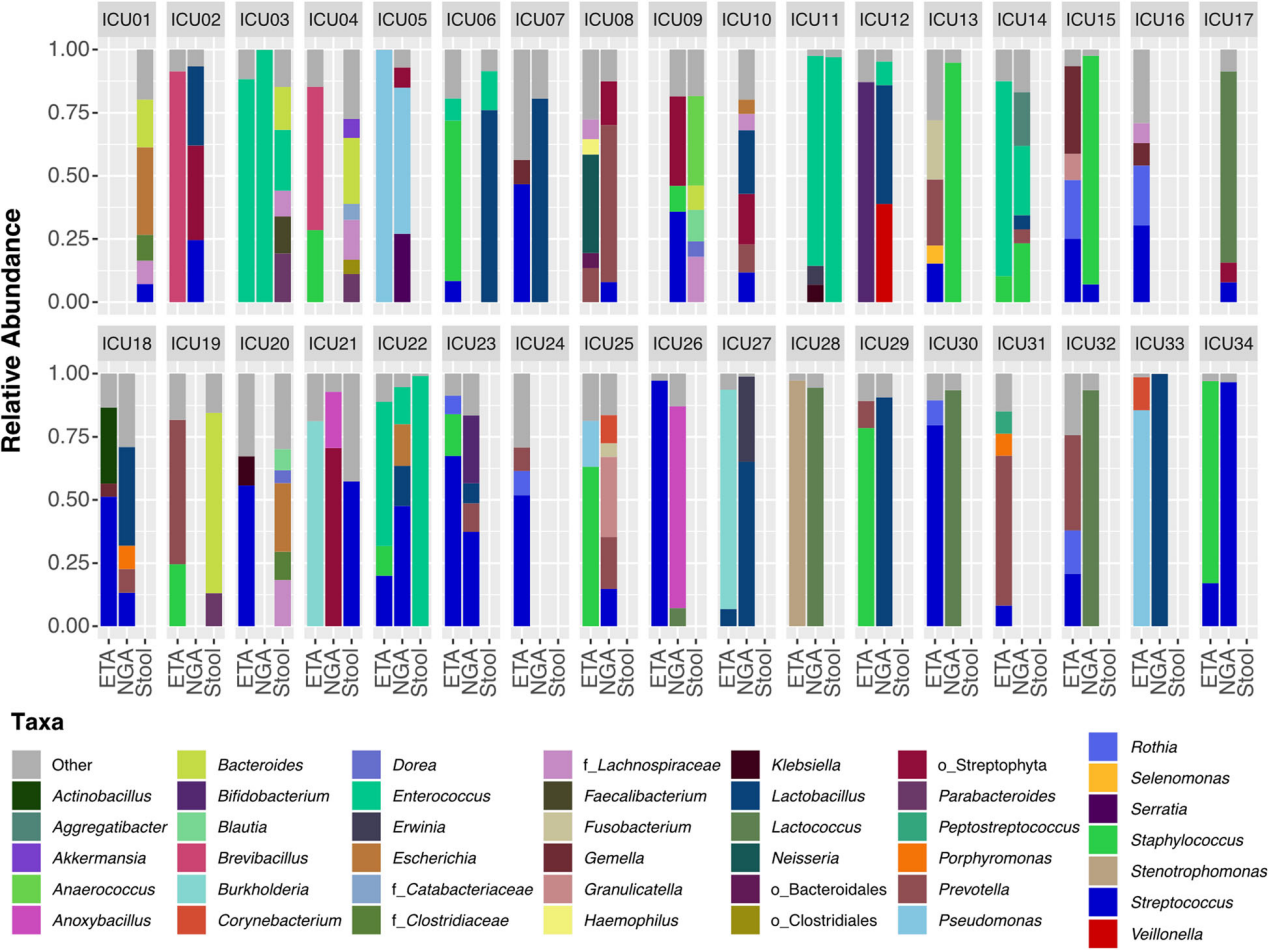
Compositional heterogeneity observed within and between anatomical sites in critically ill patients. Taxonomic summaries of the 65 samples included in this study displayed by patients and anatomical sites. Bacterial groups present at less than 5% relative abundance are grouped in the “other” category displayed in gray. Taxonomic groups are labeled according to the highest level resolved if not at the Genus (Order; o_, Family; f_)

Additionally, the hierarchical clustering of samples shows an overlap between GA and ETA and a subset of stool samples, further indicating a lack of compositional definition between anatomical sites in the ICU samples (Fig. 1c). Only the microbiota from a subset of ICU stool specimens cluster distinctly from the other samples sites (Fig. 1a, c). The heterogeneity in the microbial composition of host-associated communities within and between anatomical sites and patients is shown in the taxonomic summaries (Fig. 2). In summary, these results suggest a lack of separation between distinct anatomical sites, and high heterogeneity within body sites. Comparison with samples from a healthy cohort emphasizes the microbial dysbiosis occurring during critical illness.

### Relative abundance of specific bacterial taxa are decreased in the lower respiratory and GI tracts in the ICU cohort when compared to healthy controls

The compositional disparities between ICU and healthy specimens were investigated. We included 29 ETA samples from the ICU cohort, 7 BAL specimens from the healthy cohort, as well as 10 ICU patient stool samples and 21 healthy subject stool specimens. Analyses showed that the relative abundances of 34 OTUs for respiratory and 29 OTUs for stool samples were significantly different between critically ill vs. healthy individuals following multiple-test correction (Additional file 1: Table S3, S4). In respiratory specimens, OTUs with the greatest change in abundance were from the genera *Neisseria, Veillonella, Streptococcus, Staphylococcus,* and *Corynebacterium;* these were significantly decreased in the ICU cohort compared to the healthy cohort (Additional file 1: Table S3). OTUs from the Lachnospiraceae family, from the genera *Faecalibacterium, Blautia, Subdoligranulum*, and *Lachnobacterium* were depleted in stool specimens from ICU patients (Additional file 1: Table S4). Several of these microorganisms are members of the host-associated communities in healthy individuals [1, 39–41]. Only a few significant OTUs were increased in the ICU patients, indicating that the pathogen expansion tends to be patient-specific. However, the loss of specific bacteria from body sites was more generalized among ICU patients.

### Respiratory tract microbial diversity is associated with illness severity in the ICU cohort

**Fig. 3:**
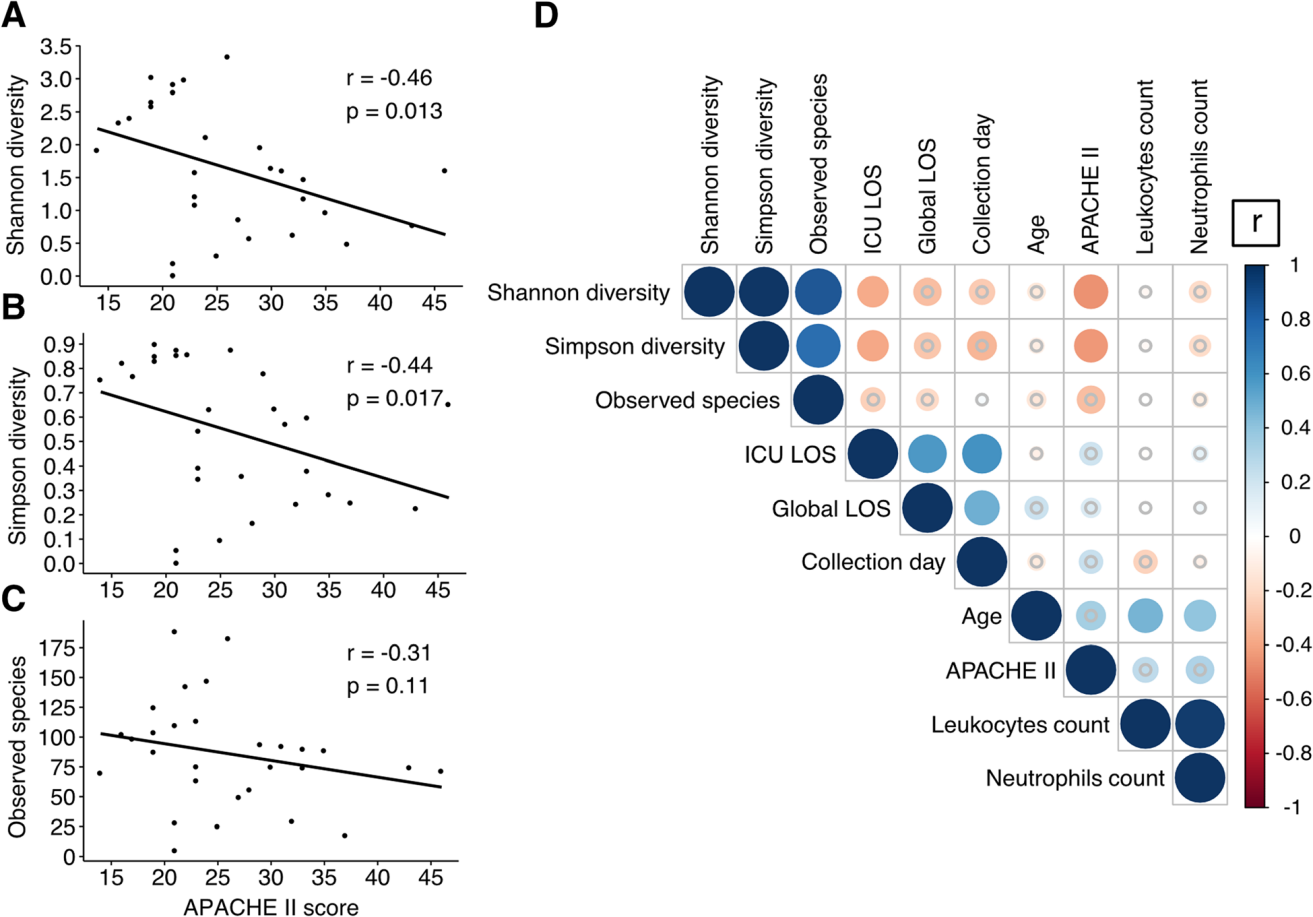
Lower respiratory tract microbial diversity is associated with illness severity in critically ill patients. Correlation analysis using Spearman’s rank correlation coefficient demonstrated an inverse association between APACHE II score and Shannon diversity (*r* = − 0.46, *p* = 0.013; **a**), Simpson diversity (*r* = − 0.44, *p* = 0.017; **b**) and Observed species (*r* = − 0.31, *p* = 0.11; **c**). Correlation matrix of clinical parameters and microbial diversity markers showed only a limited number of significant associations (**d**). The sizes of the circles are dependent on the correlation coefficient value (r). Comparisons that did not achieve significance are represented with a gray circle. LOS represents the length of stay

To determine whether microbial diversity was associated with clinical parameters, a correlation analysis was performed. Since microbial diversity and environment vary across body sites, correlation was tested separately for each anatomical site. We examined the correlation between α-diversity and APACHE II score used to assess illness severity in the ICU [42]. Both Shannon and Simpson diversity indices in ETA specimens were inversely correlated with APACHE II score (*r* = − 0.46, *p* = 0.013; Fig. 3a and r = − 0.44, *p* = 0.017; Fig. 3b, respectively). The association between APACHE II score and Observed Species was not statistically significant, but the magnitude of the correlation was similar (*r* = − 0.31, *p* = 0.11; Fig. 3c). No significant correlation between any α-diversity metric and APACHE II score was observed with GA samples (Additional file 1: Figure S2).

We also investigated the association between microbial diversity, patient demographic information, and clinical parameters in the correlation analysis (Fig. 3d). From the 45 correlation analyses included in the correlation matrix of the 29 ETA samples, 6 combinations were significant following multiple-test correction (Additional file 1: Table S5).

In summary, our results suggest a possible association between illness severity and ETA microbial diversity in mechanically ventilated patients.

### Reduced respiratory tract microbial diversity is associated with mortality in critical illness

**Fig. 4:**
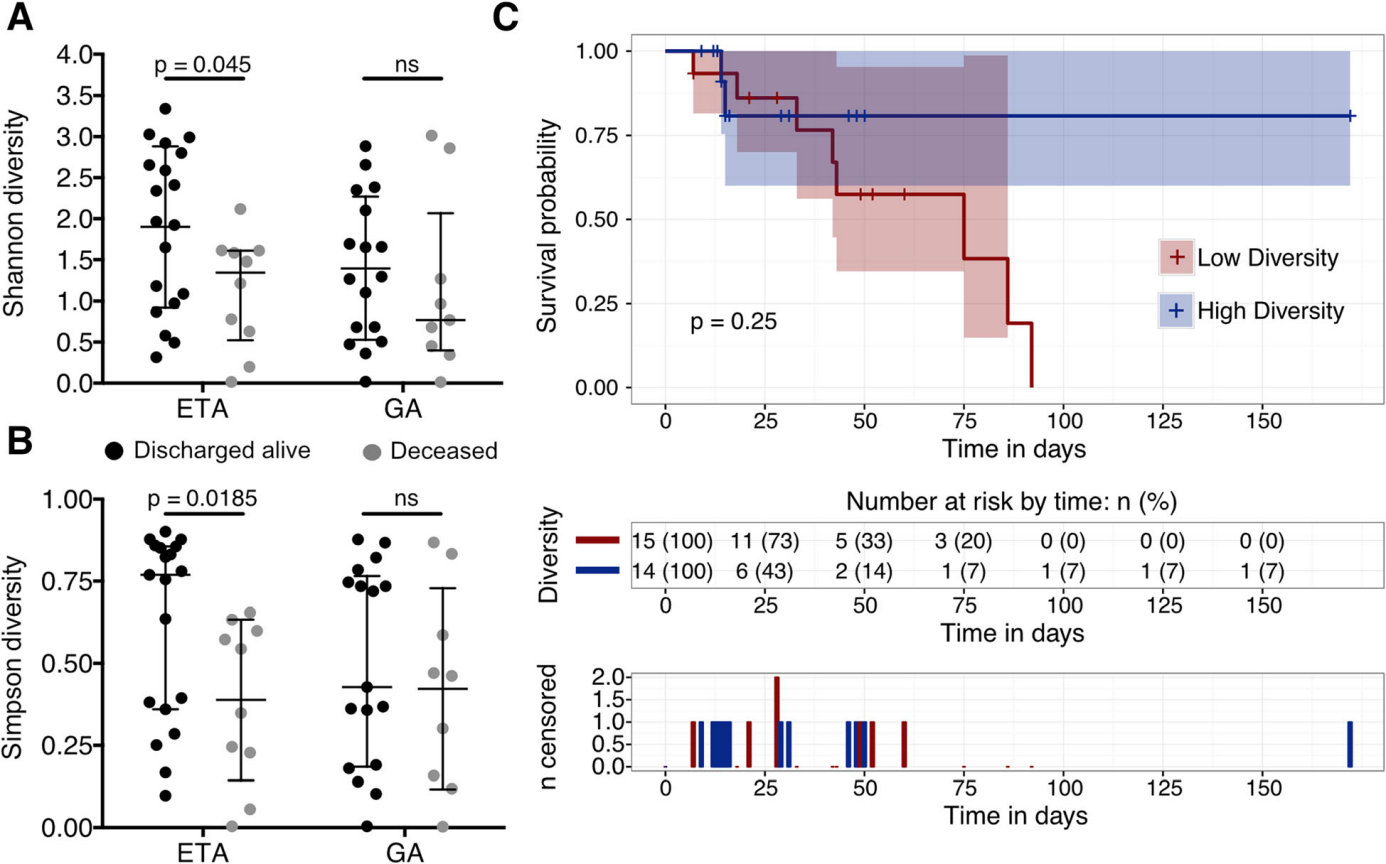
Association between microbial diversity and hospital mortality within ICU samples. Shannon (**a**) and Simpson diversity (**b**) of ETA and GA specimens shaded by hospital mortality demonstrates a significant reduction in the ETA Shannon diversity in the patients deceased in the hospital versus patients discharged alive. Kaplan-Meier survival curves displayed by high and low microbial diversity groups (**c**). The censored (i.e., discharged alive) patients are indicated by ticks marks. The threshold for grouping by diversity was the median value of the Shannon diversity measurements for the 29 samples included in this analysis. Confidence intervals are represented by the blue and red shaded areas. Numbers of patients included in the analysis and censored are shown per group under the Kaplan-Meier curve

Samples were stratified based on hospital mortality. Taxonomic summaries are included in the (Additional file 1: Figure S3). Within-site compositional differences between deceased and discharged alive patients were assessed, and no OTUs were found to be significantly different between the groups studied (Additional file 2: Table S6). However, the Shannon and Simpson diversity indices of the ETAs were significantly lower in patients who died versus those who survived their hospital stay (*p* = 0*.*045 and *p* = 0.0185; Fig. 4a, b). These results could not be explained by an increased prevalence of infection in the deceased group since both groups have a similar rate of pneumonia when specimens were collected (9/10 vs. 16/19, *p* = 1.00). Additionally, quantification of 16S rRNA gene in ETA samples demonstrated that bacterial loads were not different between both groups (*p* = 0.804; Additional file 1: Figure S4). No significant difference in ETA α-diversity was detected between admission diagnoses (Additional file 1: Figure S5). Furthermore, we failed to detect a correlation between concomitant antimicrobial exposure and Shannon (*r* = − 0.1, *p* = 0.63) and Simpson diversity indices (*r* = − 0.14, *p* = 0.5) of the ETA microbiota, nor when patients were stratified based on hospital mortality (Additional file 1: Figure S6). Moreover, specimen’s collection day was not associated with Shannon (Deceased: *r* = 0.099, *p =* 0.79; Discharged alive: *r* = − 0.15, *p =* 0.53) and Simpson diversity indices (Deceased: *r* = − 0.21, *p =* 0.56; Discharged alive: *r* = − 0.16, *p =* 0.51). GA samples did not exhibit microbial diversity differences between deceased and discharged alive patients (Fig. 4a, b), or in either anatomical site using observed species (Additional file 1: Figure S7).

We compared inflammatory markers and APACHE II score between patients with ETA who were deceased or discharged alive. No significant differences were detected in inflammatory markers (e.g. IL-6, C-reactive protein, leukocyte and neutrophil counts) or in illness severity (Additional file 1: Figure S8).

To further investigate the impact of ETA α-diversity on hospital mortality, proportions and time-to-event analyses were performed. Kaplan-Meier curves were generated to compare survival in patients with low versus high Shannon diversity. Although it appeared that patients with lower respiratory tract bacterial diversity (Shannon index < 1.61) were more likely to die in hospital over time than those with more diverse microbiota, this was not statistically significant (*p* = 0.25; Fig. 4c). From the 29 patients included in the survival analysis, 10 (34.5%) died in hospital; 2/14 (14.3%) in the high microbial diversity group died before discharge versus 8/15 (53.3%) in the low microbial diversity group (*p* = 0.05).

### Sensitivity analysis

From the 65 samples included in the study, 31 (from 23 patients) were collected before the administration of *L. rhamnosus* GG or placebo and 34 samples (from 22 patients) were collected following randomization. Our results were not affected by the removal of OTU5, assigned to *Lactobacillus*, that would included the administered probiotic *L. rhamnosus* GG or closely related indigenous strains of *Lactobacillus* from the analysis. Supplementary information concerning the results of the sensitivity analysis is available in the Additional file 1.

## Discussion

In this prospective observational study, we profiled the composition of microbial communities in a cohort of mechanically ventilated critically ill patients with a high incidence of pneumonia within their first week in the ICU, and how these communities relate to illness severity and clinical outcomes. Our results demonstrate that, in contrast to findings in healthy individuals [39], the bacterial community structures are considerably less defined by body site in the upper GI tract and lower respiratory tract. Conditions associated with admission to the ICU and interventions in this setting could compromise normal host barriers and lead to compositional overlap between the airway and stomach, reflecting a loss of microbial separation across anatomical sites in critical illness. This work builds upon early studies of the microbiome in ICU patients [7, 11, 14, 43], by including a control group with lower respiratory tract specimens to compare our findings with those of healthy individuals, and by including additional gastric samples in the ICU cohort to expand the multi-anatomical site analysis. While the proximal gastrointestinal tract is established as an important reservoir of ICU-associated pathogens [44], the role of the stomach as a source of tracheal colonization is controversial, and is not always considered to be a substantial contributor to the pathogenesis of VAP [45]. The prominent gastric colonization in ICU patients may be primarily due to use of prophylactic acid suppression to prevent stress-related gastric bleeding [46]. The overlap in microbial compositions of the respiratory tract and stomach has also been demonstrated in non-critically ill pediatric patients receiving proton-pump inhibitors [47], suggesting that these agents may contribute to the observed loss of biogeographical distinction.

The limited compositional similarities between healthy and ICU cohorts could be explained by the loss of commensal microorganisms during critical illness. We have demonstrated that ‘normal’ host-associated taxa, including the Lachnospiraceae family, *Faecalibacterium*, and *Blautia* genera in the GI tract and *Veillonella, Prevotella*, and *Neisseria* genera in the lower respiratory tract [1, 40, 41] were decreased in relative abundance in the ICU cohort. The decrease of these commensal taxa in the GI tract is consistent with other work [11, 14]. Several of the depleted OTUs are known to confer host advantages, such as anti-inflammatory and nutritional benefits via the production of short-chain fatty acid (SCFA) [3, 48, 49]. The level of fecal SCFA is drastically decreased upon admission to the ICU [9, 50]. Moreover, perturbation of the indigenous microbiota could lead to harmful repercussions and allow colonization by opportunistic secondary potential pathogens such as *C. difficile*, *Candida albicans* or facultative anaerobic gammaproteobacteria such as *Pseudomonas* [2, 51]*.* Interestingly, the OTUs that were increased in the ICU cohort in comparison to the healthy cohort were common pathogens associated with ICU-acquired infections from the *Enterococcus, Pseudomonas,* and *Staphylococcus* genera [52]. The limited number of OTUs that were significantly increased in the ICU cohort versus healthy cohort reflects the heterogeneity of the critically ill population due to various comorbidities and clinical interventions. We also demonstrate a lack of a shared microbial community structure in the ICU cohort toward a microbiota dominated by only few taxa. These results suggest that the emergence of pathogens is patient-specific, while the decrease in relative abundance of commensal taxa is observed more uniformly within the ICU population.

In this study, we demonstrated an inverse association between ETA microbial diversity and both illness severity and hospital mortality. We observed a significant decrease in respiratory microbial diversity in patients who died versus survived their hospital stay. Several studies have established that microbial composition tends to collapse in ICU patients toward the dominance of only a few taxa [12–14]. It has been suggested that the microbial collapse is driven by aggressive antimicrobial administration during critical illness. A large prospective study including 14,414 critically ill patients has demonstrated that 71% of the patients were receiving antibiotics [53]. Nevertheless, we did not see a correlation between concomitant antimicrobial exposure and ETAs microbial diversity in our cohort. This could be due to the fact that the samples included in this study were collected early during critical illness and an effect could be observed with later time points. Zakharkina et al. recently demonstrated no association between respiratory tract microbial diversity and antimicrobials in mechanically ventilated patients [8]. Moreover, it has been demonstrated that lower respiratory tract samples from mechanically ventilated patients with pneumonia tend to have lower α-diversity in comparison with patient without suspected pneumonia [15]. The loss of diversity could be due to the predominance of potential pathogens and could explain why this decrease in diversity is observed only with metrics accounting for taxa relative abundances (e.g., Shannon and Simpson diversity). However, survival groups exhibited no detectable difference of bacterial biomass nor rates of pneumonia. These results highlight the potential utilization of α-diversity metrics as an index of severity of illness and could, in addition to other clinical markers, improve patient’s stratification based on their survival prognosis. To the best of our knowledge, respiratory α-diversity has not been identified as a potential predictor of survival outcome in ICU patients. Microbial diversity of the GI tract has been associated with outcome in patients undergoing allogeneic hematopoietic stem cell transplantation, severe inflammatory response syndrome, and in high-risk patients admitted to the ICU [16, 54, 55]. By contrast, in one study of 34 ICU patients, no association between survival and microbial diversity of the GI tract was observed [17].

Our analysis was performed on samples collected during the first week of ICU stay (median collection time was 3 days following ICU admission for ETA and GA and 6 days for stool samples). This suggests that early time points could potentially be used to study the association between dysbiosis and an outcome occurring further downstream during the hospital stay (median 28.5 days IQR 14–50). Although there was no significant difference in survival curves between low and high ETA microbial diversity groups, of 29 patients included in the survival analysis, 35% died in hospital whereas 80% of the deceased patients were in the low diversity group. This suggests how ETA microbial diversity may be a complementary prognostic variable. As an indicator of illness severity akin to organ dysfunction, loss of microbial diversity can be conceptualized as a marker of poor outcome that potentially could be modifiable.

The limited number of samples and patients influences the power of these analyses. Larger studies will be required to increase our confidence in the association between microbial diversity and mortality. The comparison of critically ill patients and healthy individuals was constrained by lack of BAL specimens from the ICU cohort; therefore, more easily obtained ETA samples were used. Moreover, in clinical practice, BAL would not typically be performed early enough or commonly enough in patients without classical immunocompromised states for inclusion in our study. Although the ICU patients received several clinical interventions that could potentially affect the microbial structure of host-associated communities (e.g., antimicrobials, acid suppressants), these are intrinsic to critical care management. Adjustment for confounders such as sex, age, specific medications, and comorbidities was not suitable with our limited sample size, although no significant difference in α-diversity measurement was detected between the admission diagnoses. Moreover, despite the great heterogeneity observed in ICU patients, a proinflammatory state is a unifying feature of critical illness, regardless of the reason for admission (i.e., non-infectious conditions such as trauma and pancreatitis, as well as various infectious problems). Changes in colonization resistance and on the host’s biologic processes mediated by the microbiota may be similar across critically ill subgroups. Our study was confounded by the collection of samples over the first 7 days in the ICU. However, there was no correlation of ETAs α-diversity with time of sample collection and outcome (deceased or discharged alive).

Strengths of this study include the prospective data collection, protocolized specimen procurement and complete follow-up. We compared specimens from critically ill patients in 2 centers with a control group of healthy individuals. Our study was nested in a randomized trial testing the probiotic *L. rhamnosus* GG versus placebo; to avoid confounding results due to probiotic administration, only samples collected in the first week were included. However, we also performed a sensitivity analysis in which we removed OTU sequences assigned to *L. rhamnosus* GG and our results were not different, increasing confidence in the findings.

## Conclusions

In this study, we demonstrated that the composition of the host-associated microbial communities is severely perturbed early in mechanically ventilated critically ill patients. We found that lower respiratory tract microbial diversity is associated with illness severity and may be associated with risk of death. Together, our results suggest that critically ill patients have a potential microbiota signature associated with illness severity and hospital mortality even early in the ICU stay. The prognostic role of these microbial signatures is a promising focus for future research.

## Additional files

Additional file 1: Supplementary methodology and results. **Table S1.** ICU samples collected and additional information concerning the patients included in the study. **Table S2.** Samples collected from healthy donors. **Table S3.** OTUs significantly different in the lower respiratory tract between healthy donors and ICU patients. **Table S4.** OTUs that are significantly different in stool between healthy donors and ICU patients. **Table S5.** Correlation matrix results using Spearman rank coefficient correlation between metadata and α-diversity metrics of ETAs. **Figure S1.** Greater heterogeneity within anatomical site in the ICU cohort in comparison to a healthy cohort. **Figure S2.** Gastric microbial diversity is not associated with illness severity in critical ill patients. **Figure S3.** Microbial profiles of the ETA specimens collected from critically ill patients. **Figure S4.** Lack of association between hospital mortality and bacterial load in lower respiratory tract samples. **Figure S5.** Absence of detectable difference within microbial diversity between categories of admission. **Figure S6.** Antimicrobials exposure is not associated with ETA microbial diversity. **Figure S7.** No association between ICU samples microbial diversity and hospital mortality using the Observed Species index. **Figure S8.** Inflammatory markers and APACHE II score are not statistically different between deceased and discharged alive patients. **Figure S9**. OTU5 does not influence the loss of biogeographical distinction in ICU patients. **Figure S10.** APACHE II score association with α-diversity remains when OTU5 is removed from the analysis. **Figure S11.** OTU5 removal does not impact the decrease within microbial diversity observed in the deceased group for ETAs. **Figure S12.** Association between ICU samples microbial diversity and patient’s outcomes when OTU5 is removed. (DOCX 2905 kb)

Additional file 2: **Table S6**. Compositional differences between respiratory specimens of patients deceased versus discharged alive from the hospital. (XLS 68 kb)

## Abbreviations

BAL: Bronchoalveolar lavage
CRP: C-reactive protein
ETA: Endotracheal aspirate
FDR: False discovery rate
GA: Gastric aspirate
GI: Gastrointestinal
ICU: Intensive care unit
IL-6: Interleukin 6
NP: Nasopharynx
OP: Oropharynx
OTU: Operational taxonomic unit
SCFA: Short-chain fatty acid
VAP: Ventilator-associated pneumonia

## References

[1] Lozupone CA, Stombaugh JI, Gordon JI, Jansson JK, Knight R (2012). Diversity, stability and resilience of the human gut microbiota. Nature.

[2] Sommer F, Bäckhed F (2013). The gut microbiota--masters of host development and physiology. Nat Rev Microbiol.

[3] Levy M, Blacher E, Elinav E (2017). Microbiome, metabolites and host immunity. Curr Opin Microbiol.

[4] David LA, Materna AC, Friedman J, Campos-Baptista MI, Blackburn MC, Perrotta A (2014). Host lifestyle affects human microbiota on daily timescales. Genome Biol.

[5] Prescott HC, Dickson RP, Rogers MAM, Langa KM, Iwashyna TJ (2015). Hospitalization type and subsequent severe Sepsis. Am J Respir Crit Care Med.

[6] Kitsios GD, Morowitz MJ, Dickson RP, Huffnagle GB, McVerry BJ, Morris A (2017). Dysbiosis in the intensive care unit: microbiome science coming to the bedside. J Crit Care.

[7] Dickson RP, Singer BH, Newstead MW, Falkowski NR, Erb-Downward JR, Standiford TJ (2016). Enrichment of the lung microbiome with gut bacteria in sepsis and the acute respiratory distress syndrome. Nat Microbiol.

[8] Zakharkina T, Martin-Loeches I, Matamoros S, Povoa P, Torres A, Kastelijn JB, et al. The dynamics of the pulmonary microbiome during mechanical ventilation in the intensive care unit and the association with occurrence of pneumonia. Thorax. 2017;72:803-10. 10.1136/thoraxjnl-2016-209158 28100714

[9] Yamada T, Shimizu K, Ogura H, Asahara T, Nomoto K, Yamakawa K (2015). Rapid and sustained long-term decrease of fecal short-chain fatty acids in critically ill patients with systemic inflammatory response syndrome. JPEN J Parenter Enteral Nutr.

[10] Dickson RP (2016). The microbiome and critical illness. Lancet Respir Med.

[11] McDonald D, Ackermann G, Khailova L, Baird C, Heyland D, Kozar R, et al. Extreme Dysbiosis of the Microbiome in Critical Illness. mSphere. 2016;1:e00199–16. 10.1128/mSphere.00199-16 PMC5007431 27602409

[12] Zaborin A, Smith D, Garfield K, Quensen J, Shakhsheer B, Kade M (2014). Membership and Behavior of Ultra-Low-Diversity Pathogen Communities Present in the Gut of Humans during Prolonged Critical Illness. mBio.

[13] Berdal JE, Bjørnholt J, Blomfeldt A, Smith-Erichsen N, Bukholm G. Patterns and dynamics of airway colonisation in mechanically-ventilated patients. Clin Microbiol Infect. 2007;13:476–80. 10.1111/j.1469-0691.2006.01678.x 17430338

[14] Yeh A, Rogers MB, Firek B, Neal MD, Zuckerbraun BS, Morowitz MJ (2016). Dysbiosis across multiple body sites in critically ill adult surgical patients. Shock.

[15] Kelly BJ, Imai I, Bittinger K, Laughlin A, Fuchs BD, Bushman FD (2016). Composition and dynamics of the respiratory tract microbiome in intubated patients. Microbiome BioMed Central.

[16] Shimizu K, Ogura H, Tomono K, Tasaki O, Asahara T, Nomoto K (2010). Patterns of gram-stained fecal Flora as a quick diagnostic marker in patients with severe SIRS. Dig Dis Sci Springer US.

[17] Lankelma JM, van Vught LA, Belzer C, Schultz MJ, van der Poll T, de Vos WM (2017). Critically ill patients demonstrate large interpersonal variation in intestinal microbiota dysregulation: a pilot study. Intensive Care Med.

[18] Silvestri L, de la Cal MA, van Saene HKF (2012). Selective decontamination of the digestive tract: the mechanism of action is control of gut overgrowth. Intensive Care Med Springer-Verlag.

[19] Manzanares W, Lemieux M, Langlois PL, Wischmeyer PE (2016). Probiotic and synbiotic therapy in critical illness: a systematic review and meta-analysis. Crit Care BioMed Central.

[20] Li Q, Wang C, Tang C, He Q, Zhao X, Li N (2015). Successful treatment of severe sepsis and diarrhea after vagotomy utilizing fecal microbiota transplantation: a case report. Crit Care BioMed Central.

[21] Wei Y, Yang J, Wang J, Yang Y, Huang J, Gong H (2016). Successful treatment with fecal microbiota transplantation in patients with multiple organ dysfunction syndrome and diarrhea following severe sepsis. Crit Care BioMed Central.

[22] Cook DJ, Johnstone J, Marshall JC, Lauzier F, Thabane L, Mehta S (2016). Probiotics: Prevention of Severe Pneumonia and Endotracheal Colonization Trial—PROSPECT: a pilot trial. Trials BioMed Central.

[23] Stearns JC, Davidson CJ, McKeon S, Whelan FJ, Fontes ME, Schryvers AB (2015). Culture and molecular-based profiles show shifts in bacterial communities of the upper respiratory tract that occur with age. ISME J.

[24] Moayyedi P, Surette MG, Kim PT, Libertucci J, Wolfe M, Onischi C (2015). Fecal microbiota transplantation induces remission in patients with active ulcerative colitis in a randomized controlled trial. Gastroenterology.

[25] Potts RHG. Investigating the gut microbiome of patients with generalized anxiety disorder, major depressive disorder and bipolar patients. Master's thesis. McMaster University, Department of Medical Sciences. 2017.

[26] Baatjes AJ, Smith SG, Watson R, Howie K, Murphy D, Larché M (2015). T regulatory cell phenotypes in peripheral blood and bronchoalveolar lavage from non-asthmatic and asthmatic subjects. Clin Exp Allergy.

[27] Whelan FJ, Verschoor CP, Stearns JC, Rossi L, Luinstra K, Loeb M (2014). The loss of topography in the microbial communities of the upper respiratory tract in the elderly. Annals ATS.

[28] Whelan FJ, Surette MG (2017). A comprehensive evaluation of the sl1p pipeline for 16S rRNA gene sequencing analysis. Microbiome BioMed Central.

[29] R Core Team. R: A language and environment for statistical computing [Internet]. Vienna; 2016. http://www.R-project.org/

[30] McMurdie PJ, Holmes S. Phyloseq: an R package for reproducible interactive analysis and graphics of microbiome census data. Watson M, editor. PLoS One 2013;8:e61217–e61211. 10.1371/journal.pone.0061217 PMC3632530 23630581

[31] Morgan XC, Huttenhower C. Chapter 12: Human microbiome analysis. Lewitter F, Kann M, editors. PLoS Comput Biol. Public Libr Sci; 2012;8:e1002808. 10.1371/journal.pcbi.1002808 PMC3531975 23300406

[32] Oksanen J, Blanchet FG, Friendly M, Kindt R, Legendre P, McGlinn D, Minchin PR, O'Hara RB, Simpson GL, Solymos PH, Stevens MH, Szoecs E, Wagner H. Vegan: Community Ecology Package [Internet]. R package version 2.4–1. 2016. https://CRAN.R-project.org/package=vegan.

[33] Caporaso JG, Kuczynski J, Stombaugh J, Bittinger K, Bushman FD, Costello EK (2010). QIIME allows analysis of high-throughput community sequencing data. Nat Methods Nature Publishing Group.

[34] Harrell FE Jr, Dupont C. Hmisc: Harrell Miscellaneous [Internet]. R package version 3.17–4. 2016. https://CRAN.R-project.org/package=Hmisc.

[35] Wei T, Simko V. corrplot: Visualization of a Correlation Matrix [Internet]. R package version 0.77. 2016. https://CRAN.R-project.org/package=corrplot.

[36] Kassambara A, Kosinski M. survminer: Drawing Survival Curves using ‘ggplot2’ [Internet]. R package version 0.2.4. 2016. https://CRAN.R-project.org/package=survminer.

[37] Therneau TM. A Package for Survival Analysis in S [Internet]. R package version 2.38. 2015 https://CRAN.R-project.org/package=survival.

[38] Bates D, Mächler M (2015). Ben Bolker, Walker S. Fitting Linear Mixed-Effects Models Using lme4. J Stat Softw.

[39] The Human Microbiome Project Consortium. Structure, function and diversity of the healthy human microbiome. Nature. Nat Publ Group; 2012;486:207–214. 10.1038/nature11234 PMC3564958 22699609

[40] Charlson ES, Bittinger K, Haas AR, Fitzgerald AS, Frank I, Yadav A (2011). Topographical continuity of bacterial populations in the healthy human respiratory tract. Am J Respir Crit Care Med.

[41] Dickson RP, Erb-Downward JR, Freeman CM, McCloskey L, Falkowski NR, Huffnagle GB, et al. Bacterial Topography of the Healthy Human Lower Respiratory Tract. Clemente JC, editor. mBio American Society for Microbiology; 2017;8:e02287–e02216. 10.1128/mBio.02287-16 PMC5312084 28196961

[42] Knaus WA, Draper EA, Wagner DP, Zimmerman JE (1985). APACHE II: a severity of disease classification system. Crit Care Med.

[43] Rogers MB, Firek B, Shi M, Yeh A, Brower-Sinning R, Aveson V (2016). Disruption of the microbiota across multiple body sites in critically ill children. Microbiome BioMed Central.

[44] Marshall JC, Christou NV, Horn R, Meakins JL (1988). The microbiology of multiple organ failure. The proximal gastrointestinal tract as an occult reservoir of pathogens. Arch Surg.

[45] Chastre J, Fagon J-Y (2002). Ventilator-associated pneumonia. Am J Respir Crit Care Med.

[46] Ali T, Harty RF (2009). Stress-induced ulcer bleeding in critically ill patients. Gastroenterol Clin N Am.

[47] Rosen R, Hu L, Amirault J, Khatwa U, Ward DV, Onderdonk A (2015). 16S community profiling identifies proton pump inhibitor related differences in gastric, lung, and oropharyngeal microflora. J Pediatr.

[48] Arpaia N, Campbell C, Fan X, Dikiy S, van der Veeken J, deRoos P (2013). Metabolites produced by commensal bacteria promote peripheral regulatory T-cell generation. Nature.

[49] Sokol H, Pigneur B, Watterlot L, Lakhdari O, Bermúdez-Humarán LG, Gratadoux J-J (2008). Faecalibacterium prausnitzii is an anti-inflammatory commensal bacterium identified by gut microbiota analysis of Crohn disease patients. Proc. Natl. Acad. Sci. U.S.a. National Acad Sciences.

[50] Hayakawa M, Asahara T, Henzan N, Murakami H, Yamamoto H, Mukai N (2011). Dramatic changes of the gut flora immediately after severe and sudden insults. Dig Dis Sci.

[51] Scales BS, Dickson RP, Huffnagle GB (2016). A tale of two sites: how inflammation can reshape the microbiomes of the gut and lungs. J Leukoc Biol.

[52] Richards MJ, Edwards JR, Culver DH, Gaynes RP (2000). Nosocomial infections in combined medical-surgical intensive care units in the United States. Infect Control Hosp Epidemiol.

[53] Vincent J-L, Rello J, Marshall J, Silva E, Anzueto A, Martin CD (2009). International study of the prevalence and outcomes of infection in intensive care units. JAMA American Medical Association.

[54] Taur Y, Jenq RR, Perales M-A, Littmann ER, Morjaria S, Ling L (2014). The effects of intestinal tract bacterial diversity on mortality following allogeneic hematopoietic stem cell transplantation. Blood.

[55] Iapichino G, Callegari ML, Marzorati S, Cigada M, Corbella D, Ferrari S, et al. Impact of antibiotics on the gut microbiota of critically ill patients. J Med Microbiol. 2008;57:1007–14. 10.1099/jmm.0.47387-0 18628503

