# Supplementary material for: Microbial dysbiosis and mortality during mechanical ventilation: a prospective observational study
Source: Respir Res. 2018 Dec 7;19:245. doi: 10.1186/s12931-018-0950-5 (PMC6286574; doi:10.1186/s12931-018-0950-5)
Supplement: Supplementary file 1 — Supplementary methodology and results. Table S1. ICU samples collected and additional information concerning the patients included in the study. Table S2. Samples collected from healthy donors. Table S3. OTUs significantly different in the lower respiratory tract between healthy donors and ICU patients. Table S4. OTUs that are significantly different in stool between healthy donors and ICU patients. Table S5. Correlation matrix results using Spearman rank coefficient correlation between metadata and α-diversity metrics of ETAs. Figure S1. Greater heterogeneity within anatomical site in the ICU cohort in comparison to a healthy cohort. Figure S2. Gastric microbial diversity is not associated with illness severity in critical ill patients. Figure S3. Microbial profiles of the ETA specimens collected from critically ill patients. Figure S4. Lack of association between hospital mortality and bacterial load in lower respiratory tract samples. Figure S5. Absence of detectable difference within microbial diversity between categories of admission. Figure S6. Antimicrobials exposure is not associated with ETA microbial diversity. Figure S7. No association between ICU samples microbial diversity and hospital mortality using the Observed Species index. Figure S8. Inflammatory markers and APACHE II score are not statistically different between deceased and discharged alive patients. Figure S9. OTU5 does not influence the loss of biogeographical distinction in ICU patients. Figure S10. APACHE II score association with α-diversity remains when OTU5 is removed from the analysis. Figure S11. OTU5 removal does not impact the decrease within microbial diversity observed in the deceased group for ETAs. Figure S12. Association between ICU samples microbial diversity and patient’s outcomes when OTU5 is removed. (DOCX 2905 kb) [file 12931_2018_950_MOESM1_ESM.docx]

**Microbial Dysbiosis and Mortality During Mechanical Ventilation: A prospective observational study**

Daphnée Lamarche ^1,2,3^, Jennie Johnstone ^4,5,6^, Nicole Zytaruk ^7,8^, France Clarke ^7,8^,

Lori Hand ^8,9^, Dessi Loukov^2,10^, Jake C. Szamosi ^2,3,11^, Laura Rossi ^1,2,3^, Louis P. Schenck ^1,2,3^ (schenclp@mcmaster.ca), Chris P. Verschoor ^8^ , Ellen McDonald ^8,11^, Maureen O. Meade ^8,9,11^, John C. Marshall ^12,13^, Dawn M.E. Bowdish ^2,10^, Tim Karachi ^9,11^, Diane Heels-Ansdell ^8^, Deborah J. Cook ^7,8,11^, Michael G. Surette ^1,2,3,11^

for the PROSPECT Investigators, Canadian Critical Care Trials Group & Canadian Critical Care Translational Biology Group

**Additional File 1**

**Supplementary Methods**

***Subject recruitment***

Patients included in this study were ≥18 years of age and were expected to need mechanical ventilation for at least 72 hours. Exclusion criteria were immunocompromised patients (HIV < 200 CD4 T cells/µl, chronic immunosuppressive medications, prior organ or haematological transplant, absolute neutrophil count <500 cells/µl), patients with an increased risk of endovascular infections, patients with gastroesophageal or intestinal injury or recent surgery of the oesophagus, stomach, bowel, hepatobiliary tree, spleen or pancreas in the prior 72 hours, patients with suspected or documented ischemia, or severe acute pancreatitis, patients unable to receive enteral medications, pregnant patients, those undergoing life-support withdrawal, or patients who had been mechanically ventilated for more than 72 hours. Patients enrolled in PROSPECT were administered the study product (probiotic or placebo) twice per day via the nasogastric tube and clinical parameters and demographic data were collected.

***DNA extraction and 16S rRNA gene sequencing***

The genomic DNA extraction was performed as described in Stearns J.C., *et al.,* 2015 [1]. Briefly, 300 µl of ETA, GA or 0.1 g of fecal sample were added to a lysis buffer (800 µl of 200 mM of NaH_2_PO_4_ pH 8 and 100 µl of guanidine thyocyanate-EDTA-*N*-Lauroylsarcosine) and mechanically homogenized with 0.2 g of 0.1 mm glass beads for 3 min at 3000 rpm for ETA and GA and with 0.2 g of 2.8 mm and 0.2 g of 0.1 mm glass beads (MoBio, Carlsband, CA, USA) for 2 cycles of 3 min at 3000 rpm for fecal samples. Samples were then incubated for 1 h at 37 ˚C with 50 µL lysozyme (100 mg/ml), 50 µL mutanolysin (10 U/µl) and 10 µl RNase A (10 mg/ml) followed by 1.5 h incubation at 65 ˚C with the addition of 25 µL proteinase K (20 mg/ml), 25 µL sodium dodecyl sulfate (25%) and 75 µL NaCl (5 M). The supernatant was collected and the DNA was subsequently extracted via a phenol/chloroform extraction and purified using a Zymo Research DNA Clean and Concentrator™-25 columns (Cedarlane, Burlington, ON, Canada). The DNA was stored at -20 ˚C until further use.

Variable region 3 of the 16S rRNA gene was amplified as previously described with modest modifications [2]. Briefly, PCRs were prepared in a 50 µl reaction mixture including 200 µM of each dNTP, 1.5 mM of MgCl_2_, 5 pmol of barcoded primers and 2.5 U of Taq polymerase. Subsequently, the mixture was divided into three reactions and the PCR amplification was carried out. The PCR conditions included an initial denaturation at 94˚C for 2 min followed by 30 cycles of 94˚C for 30 sec, 50˚C for 30 sec, 72˚C for 30 sec and finished by a final extension at 72˚C for 10 min. PCR amplicons were sequenced via Illumina MiSeq Personal Sequencer system by the McMaster Genomics Facility (Hamilton, Ontario, Canada).

***Sequence processing***

The sequencing data were processed through a standardized pipeline [3]. Briefly, the sequences were trimmed of the forward and reverse primers using Cutadapt [4] and the paired-end reads were subsequently aligned using PANDAseq [5]. The alignment primers were then removed with Cutadapt [4] and low-quality reads were removed using sickle with a quality threshold of 30 (https://github.com/najoshi/sickle). Next, reads were clustered in operational taxonomic units (OTUs) using the program AbundantOTU+ [6] based on a 97% similarity threshold. Finally, taxonomy was assigned using the Ribosomal Database Project (RDP) classifier [7] against the 2011 Greengenes reference database to the genus level [8] using Quantitative Insights Into Microbial Ecology (QIIME) [9].

***Measurement of serum cytokines***

Venous blood was collected and centrifuged at 1.5 g for 10 min at 25°C and serum was stored at -140°C until processed. Serum C-reactive protein was measured by sandwich ELISA using monoclonal capture and detection antibodies (clone 5 - #ab8279, clone 6 - #ab24462; Abcam INC., Ontario, Canada) and purified human CRP as a standard (Aviva Systems Biology Corp., California, USA). Remaining serum cytokines were measured by Milliplex MAP Human High Sensitivity T Cell kit (Millipore, Ontario, Canada) as per manufacturer's protocol. Measurements were performed in duplicate and the average value was reported.

***Quantification of bacterial load***

Quantification of bacterial 16S rRNA encoding genes was performed by quantitative polymerase chain reaction (qPCR) on a Bio-Rad CFX96 thermocycler (Bio-Rad, Ontario, Canada) as previously described [10]. Briefly, reactions were carried in a 20 µL mixture including 40 ng of template, 10 pmol of each primers, 10 µg of bovine serum albumin and SsoFast EvaGreen supermix (Bio-Rad, Ontario, Canada). The qPCR program included an initial denaturation at 98˚C for 2 min followed by 40 cycles of 98˚C for 5 sec and 60˚C for 30 sec followed by the generation of the melt curve with an 0.5°C increments for 5 seconds from 65°C up to 95°C. Reactions were performed in triplicates.

***Sensitivity analysis***

OTU sequences assigned to the *Lactobacillus* genus were aligned to the *L. rhamnosus* GG 16S rRNA gene sequence using the software Geneious version 7.1.5 [11]. This was used to identify the OTU associated with the probiotic administered to patients in the probiotic group of the PROSPECT trial. To reduce downstream bias of this OTU it was removed *in silico* and the microbiota analysis was repeated.

**Supplementary Results**

***Sensitivity analysis***

Once OTU5 was removed for the sensitivity analysis, two GA samples had less than 2800 reads and were excluded from the subsequent analysis. Our main conclusions (the loss of biogeographical distinction between anatomical sites (Figure S9), an inverse correlation between ETA α diversity and APACHE II score (Figure S10), decreased ETA α diversity in patients who died in hospital (Figure S11), and the tendency of patients to have worse prognosis when they had lower respiratory microbial diversity (Figure S12) were all unaffected by the removal of OTU5. The primary difference between the two analyses lay in the significant compositional differences between stool samples from healthy controls and ICU patients, where the abundance of OTU5 was significantly increased within the ICU population (Table S4). However, We recognize that we did not eliminate or correct for the indirect impact that the probiotics administration could have systemically.

**Supplementary References**

1. Stearns JC, Davidson CJ, McKeon S, Whelan FJ, Fontes ME, Schryvers AB, et al. Culture and molecular-based profiles show shifts in bacterial communities of the upper respiratory tract that occur with age. ISME J. 2015;9:1246–59.

2. Bartram AK, Lynch MDJ, Stearns JC, Moreno-Hagelsieb G, Neufeld JD. Generation of Multimillion-Sequence 16S rRNA Gene Libraries from Complex Microbial Communities by Assembling Paired-End Illumina Reads. Appl Environ Microbiol. 2011;77:3846–52.

3. Whelan FJ, Surette MG. A comprehensive evaluation of the sl1p pipeline for 16S rRNA gene sequencing analysis. Microbiome. BioMed Central; 2017;5:100.

4. Martin M. Cutadapt removes adapter sequences from high-throughput sequencing reads. EMBnet J. 2011;17:pp.10–2.

5. Masella AP, Bartram AK, Truszkowski JM, Brown DG, Neufeld JD. PANDAseq: paired-end assembler for illumina sequences. BMC Bioinformatics 2012 13:1. BioMed Central; 2012;13:31.

6. Ye Y. Identification and quantification of abundant species from pyrosequences of 16S rRNA by consensus alignment. Proceedings (IEEE Int Conf Bioinformatics Biomed). 2010.

7. Wang Q, Garrity GM, Tiedje JM, Cole JR. Naïve Bayesian Classifier for Rapid Assignment of rRNA Sequences into the New Bacterial Taxonomy. Appl Environ Microbiol. American Society for Microbiology; 2007;73:5261–7.

8. DeSantis TZ, Hugenholtz P, Larsen N, Rojas M, Brodie EL, Keller K, et al. Greengenes, a chimera-checked 16S rRNA gene database and workbench compatible with ARB. Appl Environ Microbiol. American Society for Microbiology; 2006;72:5069–72.

9. Caporaso JG, Kuczynski J, Stombaugh J, Bittinger K, Bushman FD, Costello EK, et al. QIIME allows analysis of high-throughput community sequencing data. Nat Methods. Nature Publishing Group; 2010;7:335–6.

10. De Gregoris TB, Aldred N, Clare AS, Burgess JG. Improvement of phylum- and class-specific primers for real-time PCR quantification of bacterial taxa. J Microbiol Methods. Elsevier B.V; 2011;86:351–6.

11. Kearse M, Moir R, Wilson A, Stones-Havas S, Cheung M, Sturrock S, et al. Geneious Basic: an integrated and extendable desktop software platform for the organization and analysis of sequence data. Bioinformatics. Oxford University Press; 2012;28:1647–9.

12. Baatjes AJ, Smith SG, Watson R, Howie K, Murphy D, Larché M, et al. T regulatory cell phenotypes in peripheral blood and bronchoalveolar lavage from non-asthmatic and asthmatic subjects. Clin. Exp. Allergy. 2015;45:1654–62.

13. Moayyedi P, Surette MG, Kim PT, Libertucci J, Wolfe M, Onischi C, et al. Fecal Microbiota Transplantation Induces Remission in Patients With Active Ulcerative Colitis in a Randomized Controlled Trial. Gastroenterology. 2015;149:102–6.

14. Potts RHG, Investigating the gut microbiome of Generalized Anxiety Disorder, Major Depressive Disorder and Bipolar patients [Thesis]. 2017. Canada.

**Supplementary Figure Legends**

**Figure S1: Greater heterogeneity within anatomical site in the ICU cohort in comparison to a healthy cohort.** Tukey’ box plots of the pairwise comparisons of Bray-Curtis dissimilarity values within each types of specimens demonstrate that the microbial composition of ICU patients tends to be more variable between individuals compared to healthy individuals with respiratory and gastric specimens. The overlaying lines show the median and the interquartile range of each site. (**** indicates a *p* value < 0.001).

**Figure S2: Gastric microbial diversity is not associated with illness severity in critical ill patients.** Correlation analysis using Spearman’s rank correlation coefficient indicating that APACHE II score is not significantly correlated with Shannon (r = -0.11, *p* = 0.6; A), Simpson diversity (r = -0.089, *p* = 0.67; B) and Observed Species (r = -0.3, *p* = 0.13; C).

**Figure S3: Microbial profiles of the ETA specimens collected from critically ill patients.** Taxonomic summaries of the ETAs samples included in this study displayed by patients and hospital mortality (discharged alive or deceased). Bacterial groups present at least than 5% are grouped in the "other" category displayed in gray. Taxonomic summaries are labeled according to the highest level resolved (order; o_, family; f_).

**Figure S4: Lack of association between hospital mortality and bacterial load in lower respiratory tract samples.** Bacterial biomass estimates via quantification of the 16S rRNA gene by quantitative polymerase chain reaction failed to demonstrate a difference between patient’s deceased and discharged alive from the hospital. Measurements were performed in triplicates and the average was reported. Overlaying lines show the median and interquartile range of each group. The red dotted line represents the limit of detection. (ns refers to a *p* value > 0.05).

**Figure S5: Absence of detectable difference within microbial diversity between categories of admission.** Population proportion of deceased and discharged alive patients by category of admission (A) demonstrates that the mortality burden arises from patient with a sepsis admission diagnosis. Shannon diversity (B) and Simpson diversity (C) of ETA specimens shaded by category of admission demonstrate no detectable differences in the α diversity measurements between patients admitted due to a respiratory, sepsis or other diagnoses.

**Figure S6: Antimicrobials exposure is not associated with ETA microbial diversity.** ETA α diversity using Shannon (A-B) and Simpson diversity indexes (B-C) was not significantly correlated with concomitant antimicrobial exposure at sample collection day. Correlation analysis was performed using Spearman’s rank correlation coefficient.

**Figure S7: No association between ICU samples microbial diversity and hospital mortality using the Observed Species index.** Observed Species of ETA and GA samples demonstrated no significant reduction of the microbial diversity in the patients deceased in the hospital versus the patient discharged alive. Overlaying lines show the median and interquartile range of each group.

**Figure S8: Inflammatory markers and APACHE II score are not statistically different between deceased and discharged alive patients.** Inflammatory markers and illness severity scores were acquired from 29 patients (10 deceased and 19 discharged alive from the hospital). None of the comparisons was statistically different between the deceased and discharged alive from the hospital groups. Overlaying lines show the median and interquartile range of each group

**Figure S9: OTU5 does not influence the loss of biogeographical distinction in ICU patients.** Principal coordinates analysis **(**PCoA) using Bray-Curtis dissimilarity metric between the ICU and healthy cohorts (A) are showing that samples collected from healthy cohort tend to cluster per sample sites (B) whereas the samples from different anatomical sites tend to overlap in the ICU cohort (C).

**Figure S10: APACHE II score association with α diversity remains when OTU5 is removed from the analysis.** ETA α diversity is inversely correlated with APACHE II score using Shannon and Simpson diversity but not with Observed Species (A). This association is not observed with GA samples (B). Correlation analysis was performed using Spearman’s rank correlation coefficient.

**Figure S11: OTU5 removal does not impact the decrease within microbial diversity observed in the deceased group for ETAs.** Shannon (A), Simpson diversity (B) and Observed Species (C) of ETA and GA specimens displayed by hospital mortality demonstrated a significant reduction in the ETA microbial diversity using Shannon and Simpson diversity in the patients deceased in the hospital versus patients discharged alive.

**Figure S12: Association between ICU samples microbial diversity and patient’s outcomes when OTU5 is removed.** Kaplan-Meier survival curves displayed by microbial diversity groups showing patients censored (i.e. discharged alive) and deceased within time for the ETA samples. The threshold for the diversity group was the median value of the Shannon diversity and Simpson diversity (A) and Observed Species (B) measurements for the 29 samples included in this analysis.

**Supplementary Tables**

**Table S1: ICU samples collected and additional information concerning the patients included in the study**

| Patient ID | Samples Collection day | | | Status at discharge | ICU LOS‡ | Global LOS§ | APACHE II |
| --- | --- | --- | --- | --- | --- | --- | --- |
|  | ETA* | GA † | Stool |  |  |  |  |
| 11001 |  |  | 3 | Deceased | 16 | 332 | 39 |
| 11002 | 6 | 4 |  | Alive | 30 | 52 | 28 |
| 11003 | 5 | 3 | 5 | Deceased | 18 | 18 | 43 |
| 11004 | 2 |  | 3 | Deceased | 6 | 75 | 33 |
| 11005 | 3 | 4 |  | Deceased | 10 | 42 | 21 |
| 11008 | 6 |  | 7 | Deceased | 11 | 14 | 24 |
| 11009 | 1 | 2 |  | Alive | 3 | 14 | 22 |
| 11010 | 3 | 3 |  | Alive | 7 | 12 | 26 |
| 11011 |  | 3 | 7 | Alive | 54 | 63 | 19 |
| 11012 |  | 5 |  | Alive | 5 | 12 | 19 |
| 11014 |  | 4 | 4 | Deceased | 4 | 4 | 26 |
| 11015 | 3 | 2 |  | Alive | 18 | 49 | 35 |
| 11016 | 1 | 1 |  | Alive | 7 | 15 | 19 |
| 11017 | 2 | 2 |  | Alive | 4 | 28 | 23 |
| 11018 | 4 | 2 |  | Alive | 21 | 46 | 29 |
| 11019 | 1 |  |  | Alive | 4 | 13 | 21 |
| 11020 |  | 2 |  | Alive | 5 | 5 | 6 |
| 11022 | 3 | 6 |  | Deceased | 15 | 15 | 46 |
| 11023 | 4 |  | 6 | Alive | 12 | 48 | 16 |
| 11024 | 3 |  | 6 | Alive | 12 | 172 | 19 |
| 11025 | 7 | 4 | 7 | Deceased | 34 | 43 | 23 |
| 11026 | 3 | 3 | 6 | Alive | 7 | 50 | 30 |
| 11027 | 2 | 2 |  | Alive | 22 | 29 | 14 |
| 21001 | 2 |  |  | Alive | 6 | 9 | 17 |
| 21002 | 3 | 3 |  | Deceased | 86 | 86 | 31 |
| 21003 | 2 | 2 |  | Alive | 7 | 7 | 25 |
| 21004 | 4 | 4 |  | Deceased | 9 | 33 | 32 |
| 21005 | 5 | 5 |  | Deceased | 73 | 92 | 21 |
| 21006 | 7 | 4 |  | Alive | 60 | 60 | 33 |
| 21008 | 2 | 2 |  | Deceased | 7 | 7 | 23 |
| 21009 | 3 |  |  | Alive | 31 | 31 | 19 |
| 21010 | 3 | 3 |  | Alive | 7 | 16 | 21 |
| 21011 | 1 | 4 |  | Alive | 19 | 28 | 37 |
| 21012 | 2 | 4 |  | Alive | 15 | 21 | 27 |

*ETA: endotracheal tube aspirate †GA: gastric tube aspirate ‡LOS: Length of stay

§Global LOS: represents the days between ICU admission and hospital discharge

**Table S2: Samples collected from healthy donors**

| Specimen's type | Donors (n) | Sample's ID | Age | Sex | Reference |
| --- | --- | --- | --- | --- | --- |
| BAL | 7 | BAL1 | NA | NA | [12] |
|  |  | BAL2 | NA | NA |  |
|  |  | BAL3 | NA | NA |  |
|  |  | BAL4 | NA | NA |  |
|  |  | BAL5 | NA | NA |  |
|  |  | BAL6 | NA | NA |  |
|  |  | BAL7 | NA | NA |  |
| NP swab | 7 | NP1 | NA | Female | [1] |
|  |  | NP2 | NA | Female |  |
|  |  | NP3 | NA | NA |  |
|  |  | NP4 | NA | NA |  |
|  |  | NP5 | NA | Male |  |
|  |  | NP6 | NA | Male |  |
|  |  | NP7 | NA | Male |  |
| OP swab | 7 | OP1 | NA | Female |  |
|  |  | OP2 | NA | Female |  |
|  |  | OP3 | NA | NA |  |
|  |  | OP4 | NA | NA |  |
|  |  | OP5 | NA | Male |  |
|  |  | OP6 | NA | Male |  |
|  |  | OP7 | NA | Male |  |
| Stool | 21 | S1 | NA | NA | [13] |
|  |  | S2 | NA | NA |  |
|  |  | S3 | NA | NA |  |
|  |  | S4 | NA | NA |  |
|  |  | S5 | NA | NA |  |
|  |  | S6 | NA | NA |  |
|  |  | S7 | 40 | Male | [14] |
|  |  | S8 | 25 | Male |  |
|  |  | S9 | 39 | Female |  |
|  |  | S10 | 55 | Female |  |
|  |  | S11 | 40 | Male |  |
|  |  | S12 | 29 | Male |  |
|  |  | S13 | 20 | Female |  |
|  |  | S14 | 23 | Male |  |
|  |  | S15 | 60 | Female |  |
|  |  | S16 | 54 | Female |  |
|  |  | S17 | 29 | Female |  |
|  |  | S18 | 39 | Male |  |
|  |  | S19 | 64 | Male |  |
|  |  | S20 | 39 | Female |  |
|  |  | S21 | 24 | Female |  |

**Table S3: OTUs significantly different in the lower respiratory tract between healthy donors and ICU patients.**

| Taxonomy | | | Relative abundance | | | *p* value (after FDR) |
| --- | --- | --- | --- | --- | --- | --- |
| Order | Family | Genus | Healthy | ICU | Δ |  |
| Neisseriales | Neisseriaceae | *Neisseria* | 36.26 | 1.30 | 34.95 | 0.001 |
| Clostridiales | Veillonellaceae | *Veillonella* | 3.34 | 0.18 | 3.16 | 0.001 |
| Lactobacillales | Streptococcaceae | *Streptococcus* | 2.81 | 0.11 | 2.70 | < 0.001 |
| Bacillales | Staphylococcaceae | *Staphylococcus* | 2.40 | 0.08 | 2.32 | < 0.001 |
| Actinomycetales | Corynebacteriaceae | *Corynebacterium* | 2.23 | 0.03 | 2.21 | < 0.001 |
| Clostridiales | Veillonellaceae | *Megasphaera* | 1.44 | 0.11 | 1.33 | < 0.001 |
| Pasteurellales | Pasteurellaceae | *Haemophilus* | 0.88 | 0.18 | 0.70 | 0.003 |
| Caulobacterales | Caulobacteraceae | *Caulobacter* | 0.67 | 0.01 | 0.67 | < 0.001 |
| Clostridiales | Veillonellaceae | *Veillonella* | 0.61 | 0.04 | 0.56 | < 0.001 |
| Clostridiales | Veillonellaceae | *Selenomonas* | 0.36 | 0.06 | 0.29 | 0.001 |
| Fusobacteriales | Fusobacteriaceae | *Fusobacterium* | 0.22 | 0.00 | 0.22 | 0.003 |
| Bacteroidales | Prevotellaceae | *Prevotella* | 0.19 | 0.00 | 0.19 | < 0.001 |
| Enterobacteriales | Enterobacteriaceae | *Proteus* | 0.19 | 0.00 | 0.19 | < 0.001 |
| Bacteroidales | Prevotellaceae | *Prevotella* | 0.18 | 0.01 | 0.17 | 0.008 |
| Actinomycetales | Actinomycetaceae | *Actinomyces* | 0.20 | 0.06 | 0.14 | 0.031 |
| Sphingomonadales | Sphingomonadaceae |  | 0.14 | 0.02 | 0.12 | 0.003 |
| Burkholderiales | Comamonadaceae | *Caldimonas* | 0.12 | 0.00 | 0.12 | < 0.001 |
| Pseudomonadales | Pseudomonadaceae | *Pseudomonas* | 0.10 | 0.00 | 0.10 | < 0.001 |
| Pseudomonadales | Pseudomonadaceae | *Pseudomonas* | 0.09 | 0.00 | 0.09 | 0.003 |
| Clostridiales | ClostridialesFamilyXIII.IncertaeSedis | *Eubacterium* | 0.10 | 0.02 | 0.08 | 0.012 |
| Actinomycetales |  |  | 0.14 | 0.07 | 0.08 | 0.006 |
| Clostridiales | Veillonellaceae | *Veillonella* | 0.08 | 0.00 | 0.08 | 0.002 |
| Clostridiales | Veillonellaceae |  | 0.07 | 0.00 | 0.07 | 0.028 |
| Fusobacteriales | Fusobacteriaceae | *Fusobacterium* | 0.07 | 0.01 | 0.06 | 0.045 |
| Sphingomonadales | Sphingomonadaceae | *Novosphingobium* | 0.06 | 0.00 | 0.06 | < 0.001 |
| Clostridiales | Veillonellaceae | *Selenomonas* | 0.08 | 0.03 | 0.05 | 0.035 |
| Erysipelotrichales | Erysipelotrichaceae | *Bulleidia* | 0.11 | 0.07 | 0.03 | 0.040 |
| Neisseriales | Neisseriaceae | *Neisseria* | 0.08 | 0.05 | 0.03 | 0.040 |
| Fusobacteriales | Fusobacteriaceae | *Fusobacterium* | 1.09 | 1.07 | 0.02 | 0.013 |
| Lactobacillales | Streptococcaceae | *Streptococcus* | 1.00 | 2.05 | -1.05 | 0.048 |
| Mycoplasmatales | Mycoplasmataceae | *Mycoplasma* | 0.00 | 0.26 | -0.26 | 0.012 |
| Clostridiales | Peptostreptococcaceae | *Peptostreptococcus* | 0.42 | 0.56 | -0.14 | 0.029 |
| Clostridiales | Veillonellaceae | *Selenomonas* | 0.03 | 0.05 | -0.02 | 0.005 |
| Neisseriales | Neisseriaceae | *Neisseria* | 0.16 | 0.17 | -0.02 | 0.044 |

**Table S4: OTUs that are significantly different in stool between healthy donors and ICU patients.**

| Taxonomy | | | Relative abundance | | | *p* value (after FDR) |
| --- | --- | --- | --- | --- | --- | --- |
| Order | Family | Genus | Healthy | ICU | Δ |  |
| Clostridiales | Lachnospiraceae |  | 28.71 | 6.83 | 21.88 | 0.003 |
| Clostridiales | Ruminococcaceae | *Faecalibacterium* | 8.41 | 1.53 | 6.88 | 0.005 |
| Clostridiales | Lachnospiraceae | *Blautia* | 7.95 | 2.60 | 5.35 | 0.030 |
| Clostridiales | Lachnospiraceae |  | 2.81 | 0.13 | 2.68 | 0.003 |
| Clostridiales | Ruminococcaceae | *Subdoligranulum* | 1.19 | 0.07 | 1.12 | 0.004 |
| Clostridiales | Lachnospiraceae | *Lachnobacterium* | 1.02 | 0.01 | 1.01 | 0.002 |
| Clostridiales | Ruminococcaceae | *Oscillospira* | 0.99 | 0.03 | 0.96 | 0.004 |
| Coriobacteriales | Coriobacteriaceae | *Collinsella* | 0.37 | 0.02 | 0.35 | 0.013 |
| Clostridiales |  |  | 0.40 | 0.06 | 0.34 | 0.045 |
| Clostridiales | Lachnospiraceae | *Shuttleworthia* | 0.33 | 0.01 | 0.32 | 0.042 |
| Clostridiales | Ruminococcaceae | *Clostridium* | 0.28 | 0.00 | 0.28 | 0.013 |
| Clostridiales | Ruminococcaceae | *Oscillospira* | 0.30 | 0.04 | 0.26 | 0.042 |
| Erysipelotrichales | Erysipelotrichaceae | *Clostridium* | 0.26 | 0.00 | 0.26 | 0.013 |
| Clostridiales |  |  | 0.25 | 0.00 | 0.25 | 0.003 |
| Clostridiales | Lachnospiraceae |  | 0.31 | 0.07 | 0.24 | 0.005 |
| Clostridiales |  |  | 0.24 | 0.03 | 0.21 | 0.004 |
| Clostridiales |  |  | 0.19 | 0.00 | 0.19 | 0.030 |
| Clostridiales | Lachnospiraceae | *Anaerostipes* | 0.21 | 0.04 | 0.17 | 0.029 |
| Clostridiales |  |  | 0.16 | 0.00 | 0.16 | 0.023 |
| Clostridiales | Ruminococcaceae |  | 0.15 | 0.03 | 0.13 | 0.021 |
| Clostridiales | Lachnospiraceae | *Ruminococcus* | 0.11 | 0.01 | 0.10 | 0.020 |
| Lactobacillales | Enterococcaceae | *Enterococcus* | 0.00 | 23.39 | -23.39 | 0.002 |
| Lactobacillales | Lactobacillaceae | *Lactobacillus* | 0.00 | 7.87 | -7.87 | 0.000 |
| Erysipelotrichales | Erysipelotrichaceae |  | 0.01 | 0.54 | -0.53 | 0.025 |
| Bacillales | Staphylococcaceae | *Staphylococcus* | 0.00 | 0.53 | -0.53 | 0.004 |
| Erysipelotrichales | Erysipelotrichaceae | *Clostridium* | 0.01 | 0.39 | -0.38 | 0.020 |
| Lactobacillales | Enterococcaceae | *Enterococcus* | 0.00 | 0.11 | -0.11 | 0.030 |
| Bacteroidales | Porphyromonadaceae | *Parabacteroides* | 0.00 | 0.05 | -0.05 | 0.030 |
| Pseudomonadales | Pseudomonadaceae | *Pseudomonas* | 0.00 | 0.04 | -0.03 | 0.032 |

**Table S5: Correlation matrix results using Spearman rank coefficient correlation between metadata and α diversity metrics of ETAs**

| Variable 1 | Variable 2 | β coefficient | *p* value | |
| --- | --- | --- | --- | --- |
|  |  |  | No FDR | With FDR |
| Age | Collection time (ICU days) | -0.12 | 0.535 | 0.730 |
| Age | Leukocytes count | 0.46 | 0.015 | 0.068 |
| Age | Neutrophils count | 0.36 | 0.062 | 0.216 |
| APACHE II score | Age | 0.34 | 0.072 | 0.229 |
| APACHE II score | Collection time (ICU days) | 0.22 | 0.248 | 0.486 |
| APACHE II score | Leukocytes count | 0.22 | 0.253 | 0.455 |
| APACHE II score | Neutrophils count | 0.26 | 0.185 | 0.396 |
| Collection time (ICU days) | Leukocytes count | -0.24 | 0.210 | 0.430 |
| Collection time (ICU days) | Neutrophils count | -0.12 | 0.563 | 0.745 |
| Global Length of stay | Age | 0.21 | 0.264 | 0.496 |
| Global Length of stay | APACHE II score | 0.15 | 0.427 | 0.686 |
| Global Length of stay | Collection time (ICU days) | 0.48 | 0.008 | 0.053 |
| Global Length of stay | ICU Length of stay | 0.57 | 0.001 | 0.009 |
| Global Length of stay | Leukocytes count | -0.03 | 0.697 | 0.829 |
| Global Length of stay | Neutrophils count | 0.00 | 0.998 | 0.998 |
| Global Length of stay | Observed Species | -0.19 | 0.319 | 0.553 |
| Global Length of stay | Shannon diversity | -0.30 | 0.113 | 0.283 |
| Global Length of stay | Simpson diversity | -0.28 | 0.148 | 0.351 |
| ICU Length of stay | Age | -0.08 | 0.674 | 0.843 |
| ICU Length of stay | APACHE II score | 0.21 | 0.284 | 0.492 |
| ICU Length of stay | Collection time (ICU days) | 0.61 | < 0.001 | 0.004 |
| ICU Length of stay | Leukocytes count | -0.04 | 0.829 | 0.949 |
| ICU Length of stay | Neutrophils count | 0.08 | 0.710 | 0.860 |
| Neutrophils count | Leukocytes count | 0.96 | < 0.001 | < 0.001 |
| Observed Species | Age | -0.14 | 0.471 | 0.687 |
| Observed Species | APACHE II score | -0.31 | 0.107 | 0.282 |
| Observed Species | Collection time (ICU days) | 0.04 | 0.843 | 0.949 |
| Observed Species | ICU Length of stay | -0.23 | 0.220 | 0.430 |
| Observed Species | Leukocytes count | 0.02 | 0.928 | 0.989 |
| Observed Species | Neutrophils count | -0.07 | 0.726 | 0.860 |
| Observed Species | Shannon diversity | 0.85 | < 0.001 | < 0.001 |
| Observed Species | Simpson diversity | 0.77 | < 0.001 | < 0.001 |
| Shannon diversity | Age | -0.12 | 0.523 | 0.730 |
| Shannon diversity | APACHE II score | -0.46 | 0.013 | 0.082 |
| Shannon diversity | Collection time (ICU days) | -0.26 | 0.174 | 0.412 |
| Shannon diversity | ICU Length of stay | -0.38 | 0.045 | 0.167 |
| Table continues next page | | | | |
| Variable 1 | Variable 2 | β coefficient | *p* value | |
|  |  |  | No FDR | With FDR |
| Shannon diversity | Leukocytes count | -0.03 | 0.897 | 0.982 |
| Shannon diversity | Neutrophils count | -0.14 | 0.471 | 0.687 |
| Simpson diversity | Age | -0.10 | 0.624 | 0.802 |
| Simpson diversity | APACHE II score | -0.44 | 0.017 | 0.078 |
| Simpson diversity | Collection time (ICU days) | -0.33 | 0.076 | 0.229 |
| Simpson diversity | ICU Length of stay | -0.38 | 0.041 | 0.167 |
| Simpson diversity | Leukocytes count | -0.01 | 0.953 | 0.982 |
| Simpson diversity | Neutrophils count | -0.14 | 0.473 | 0.687 |
| Simpson diversity | Shannon diversity | 0.98 | < 0.001 | < 0.001 |

Global length of stay is the number of days between ICU admission and hospital discharge

FDR: False discovery rate

**Table S6: Compositional differences between respiratory specimens of patients deceased versus discharged alive from the hospital.**

The table is uploaded as Additional file 2


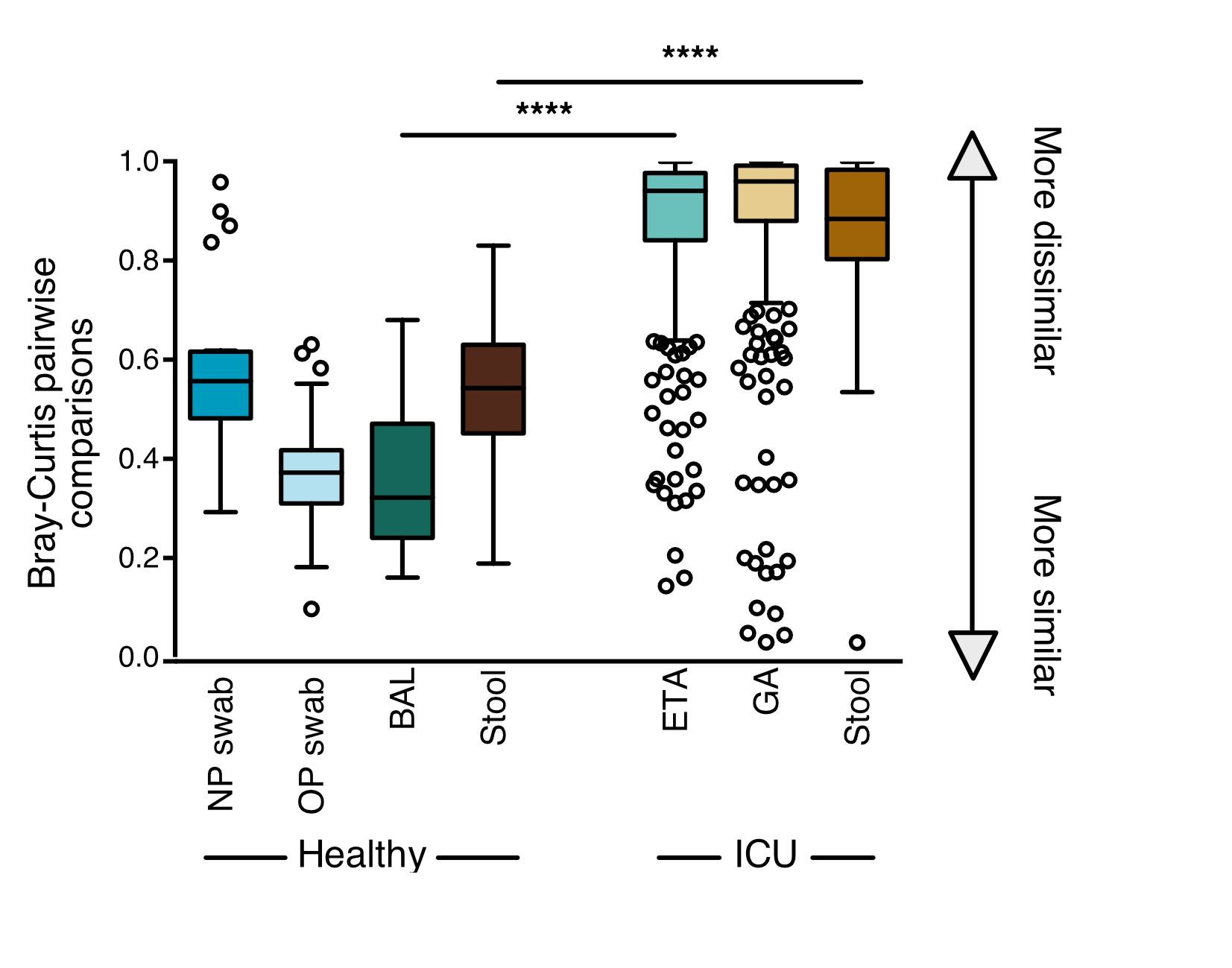


Figure S1


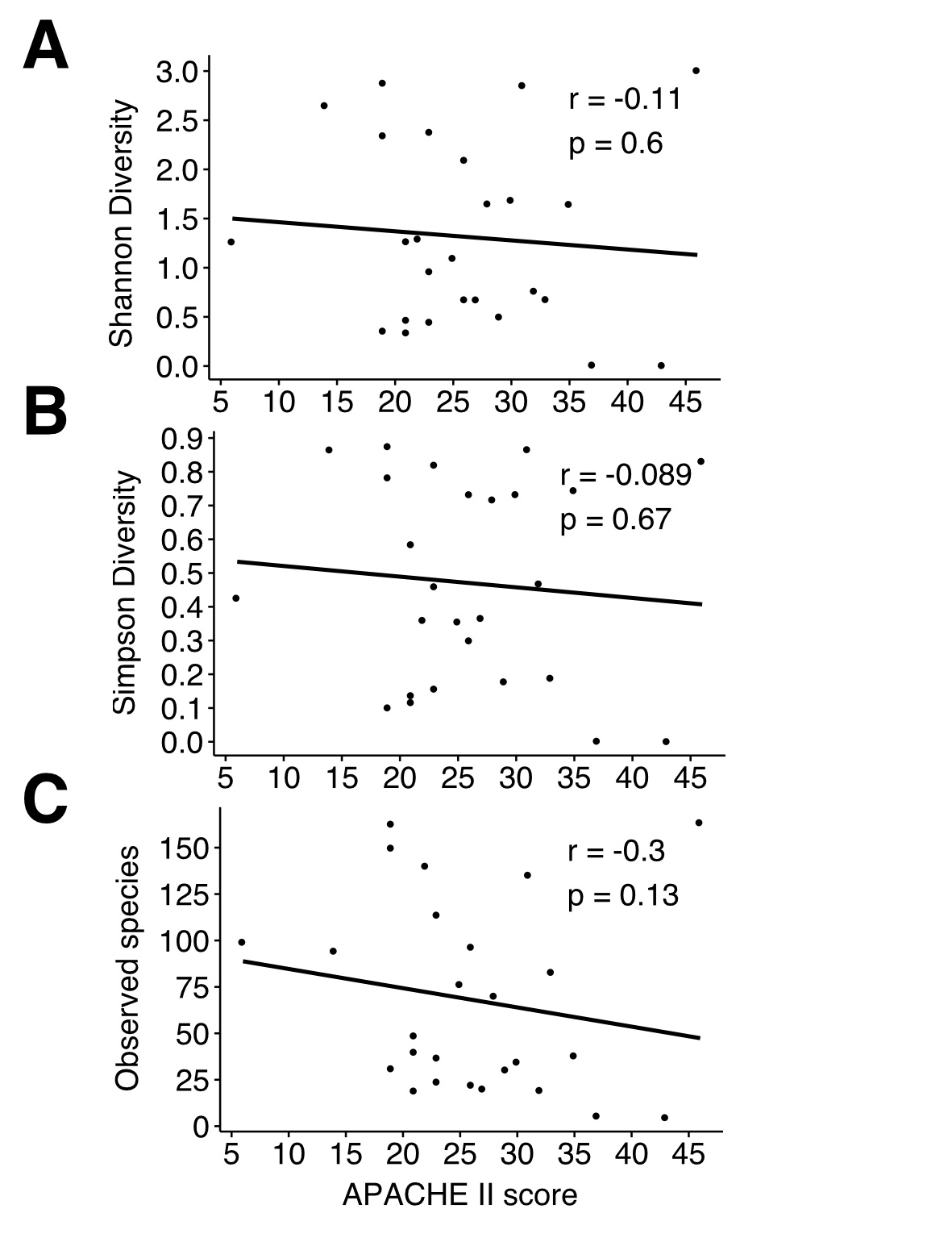


Figure S2


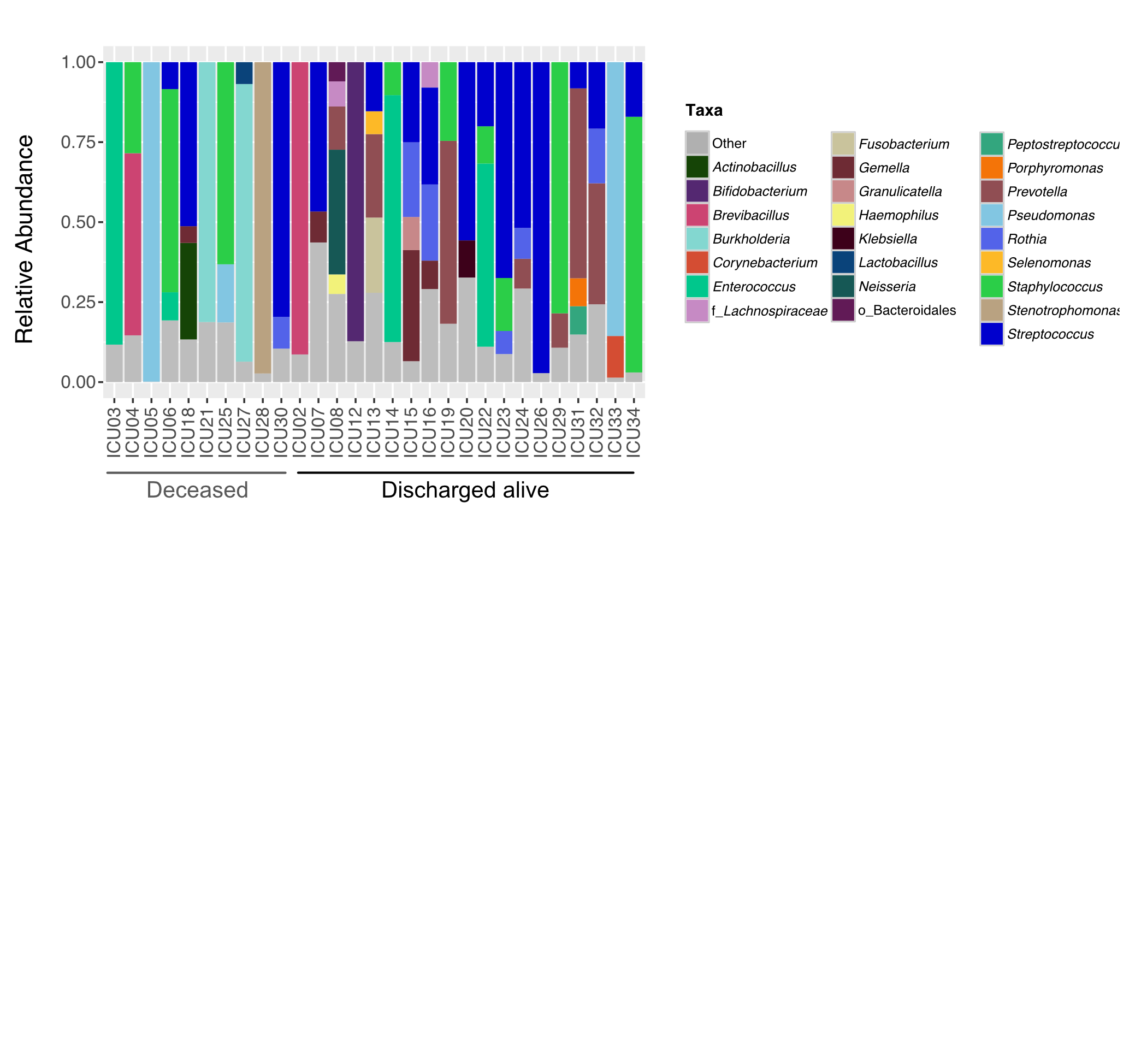


Figure S3

Figure S4


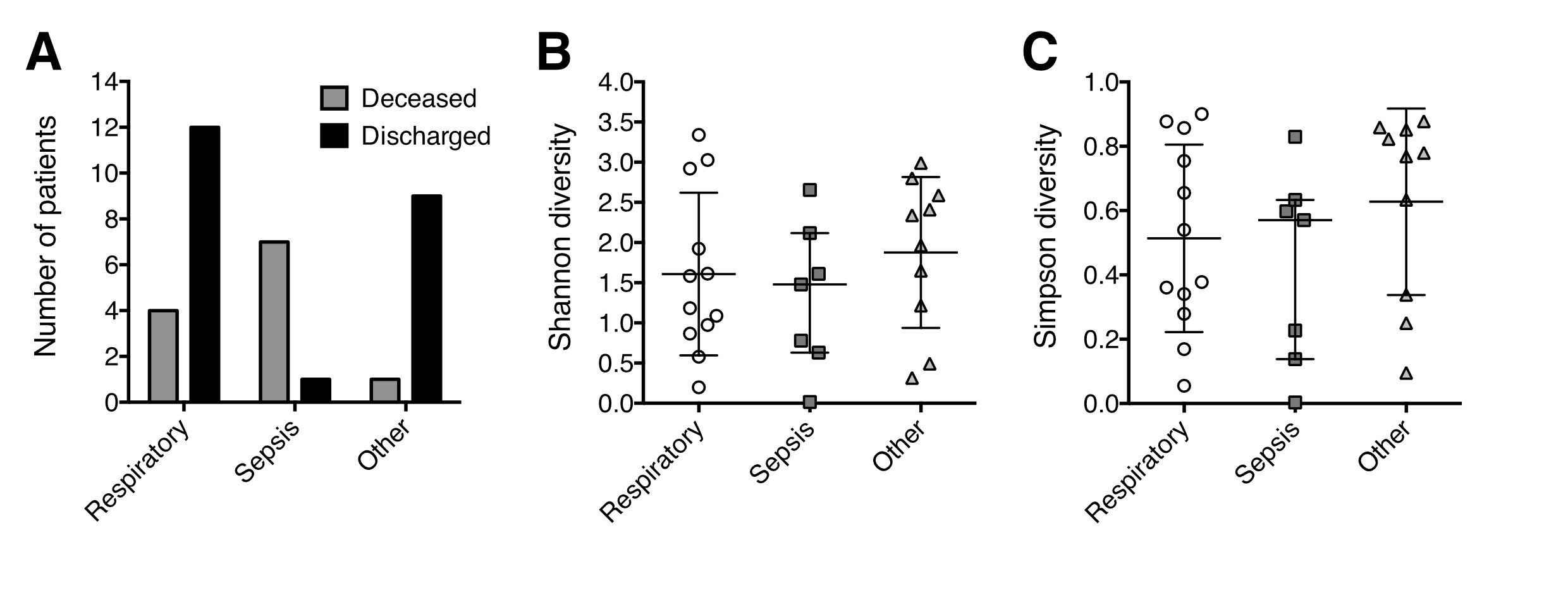


Figure S5


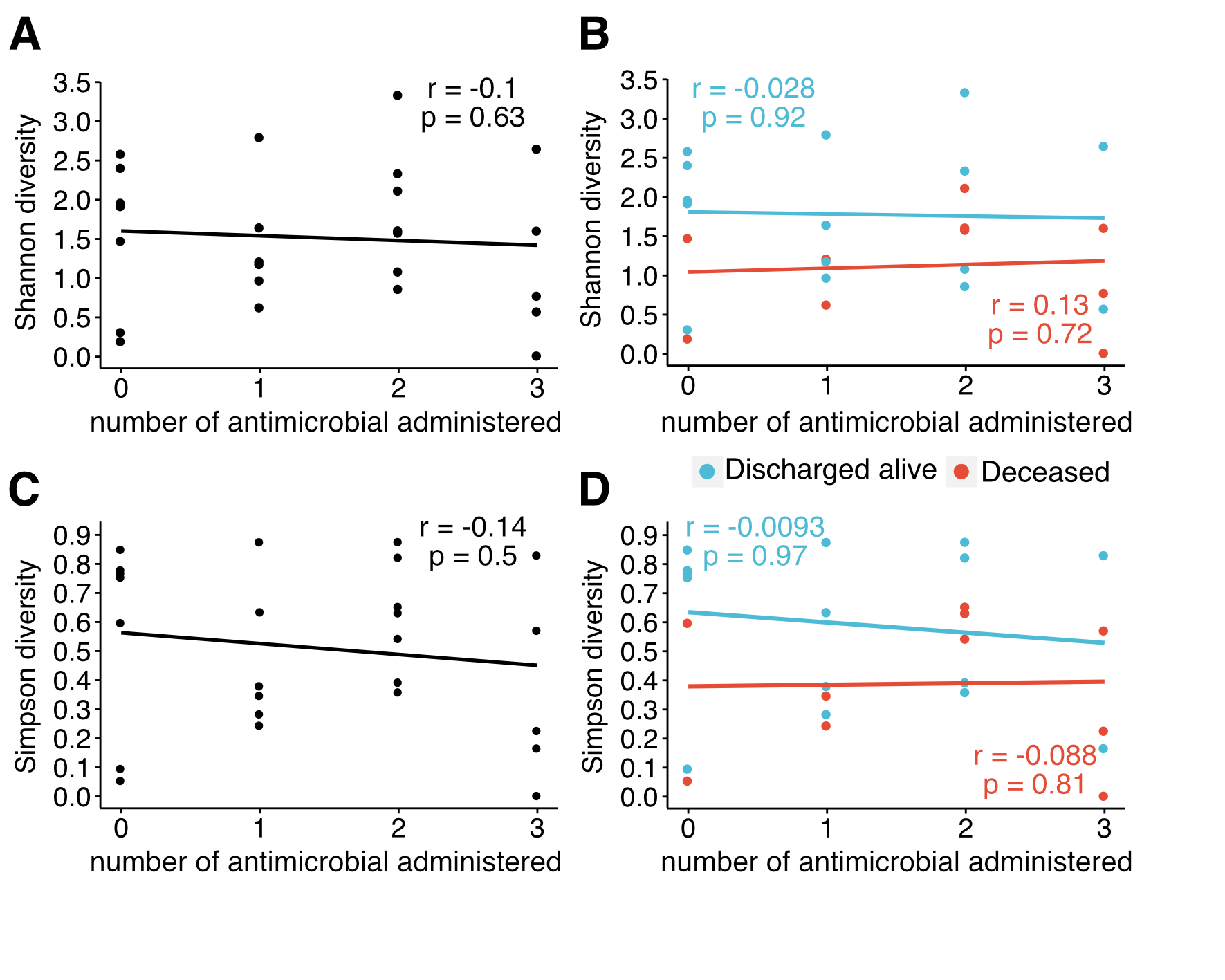


Figure S6


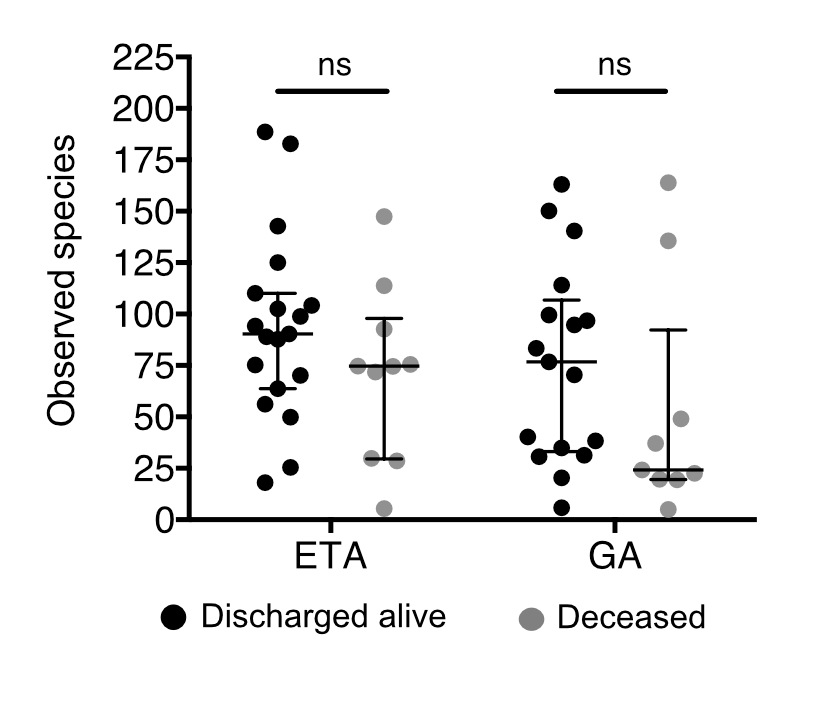


Figure S7

Figure S8


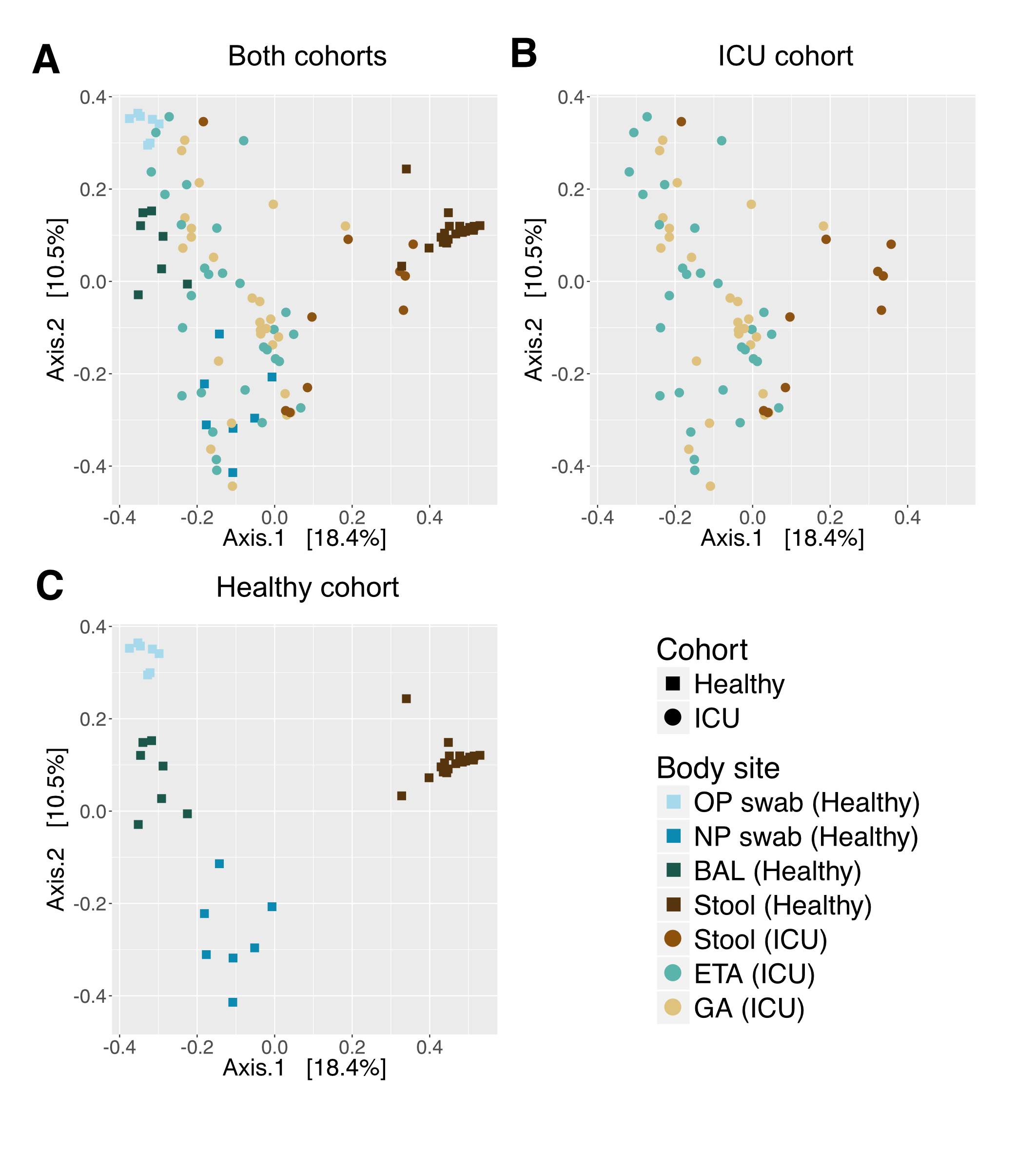


Figure S9


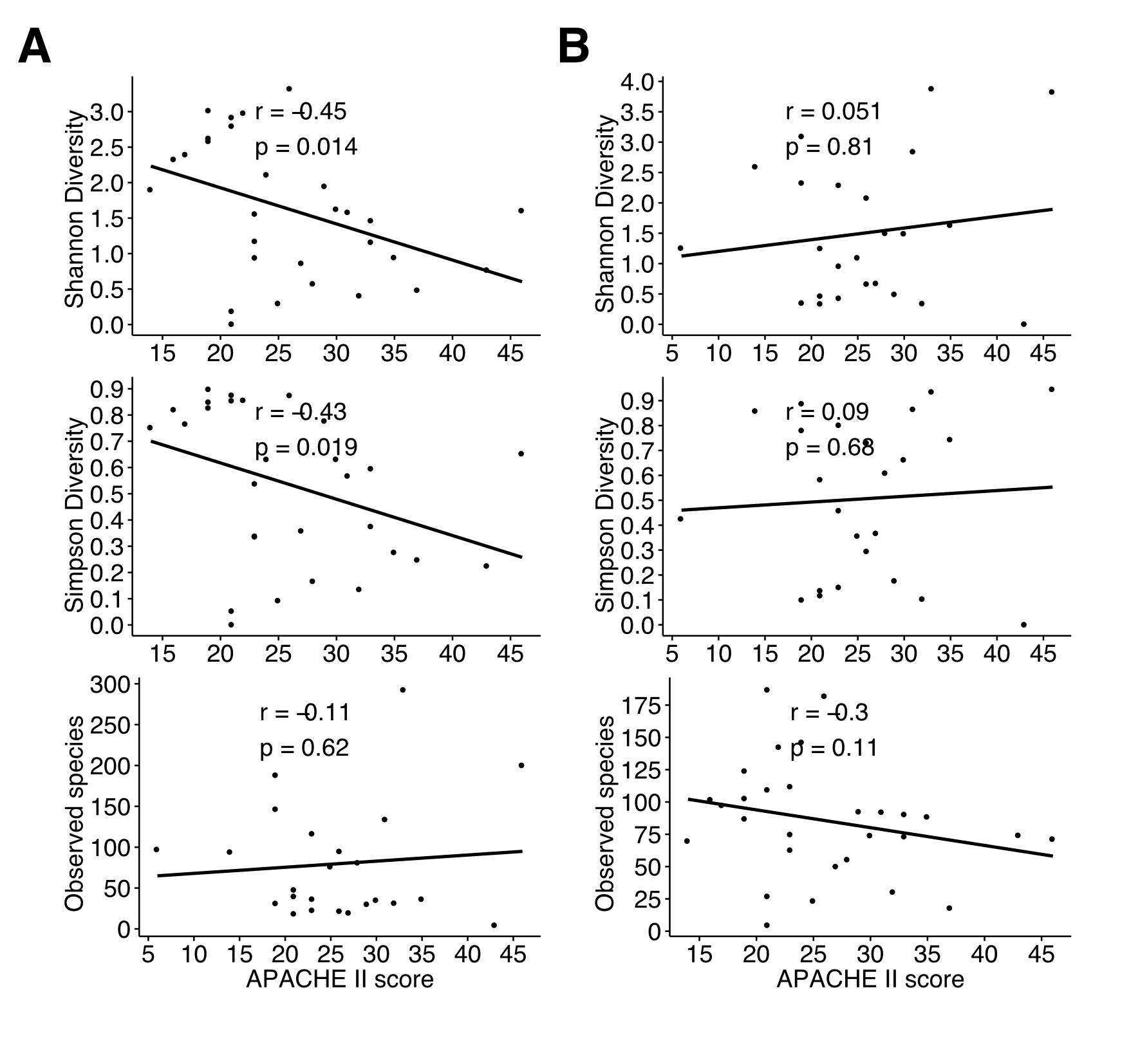


Figure S10


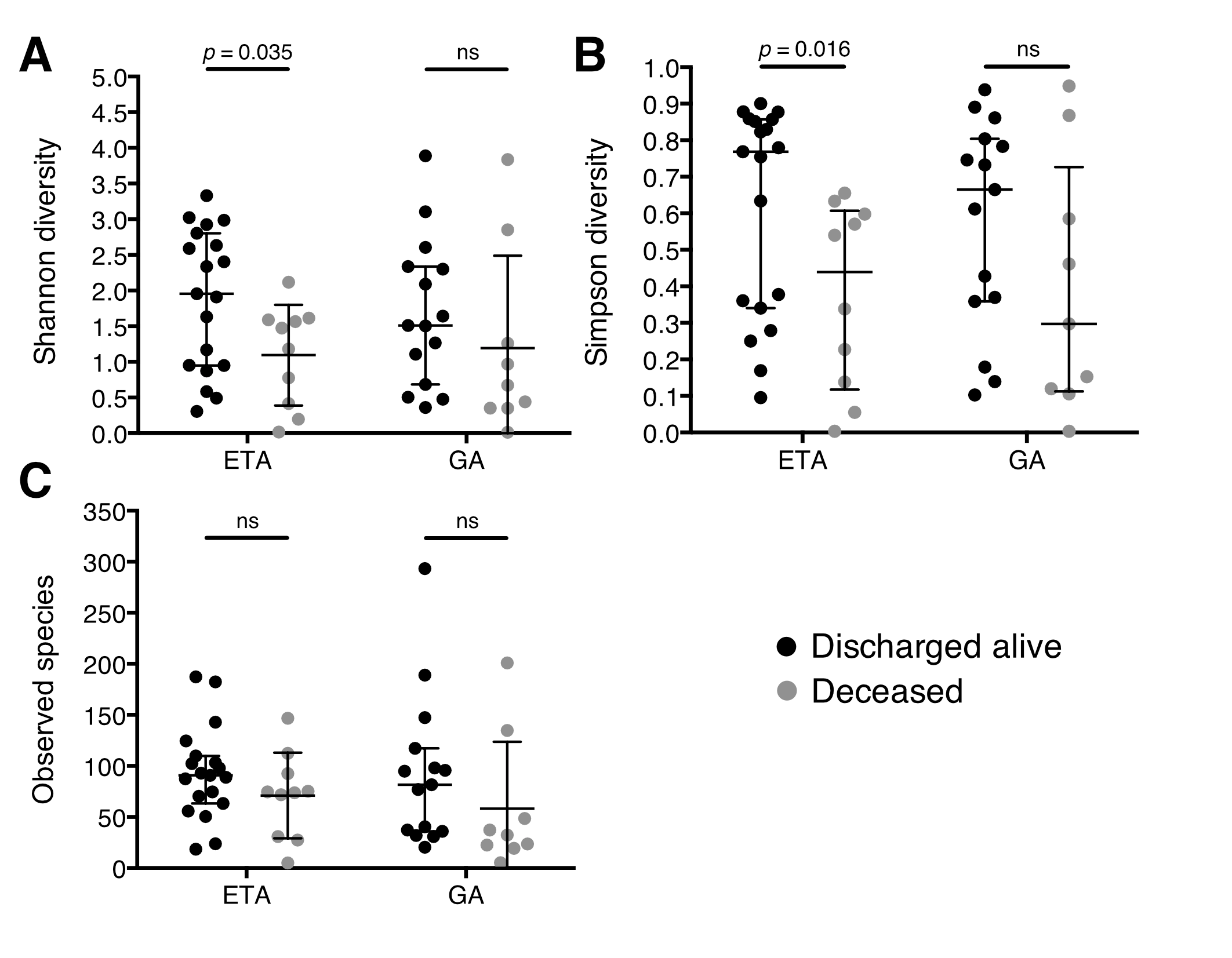


Figure S11


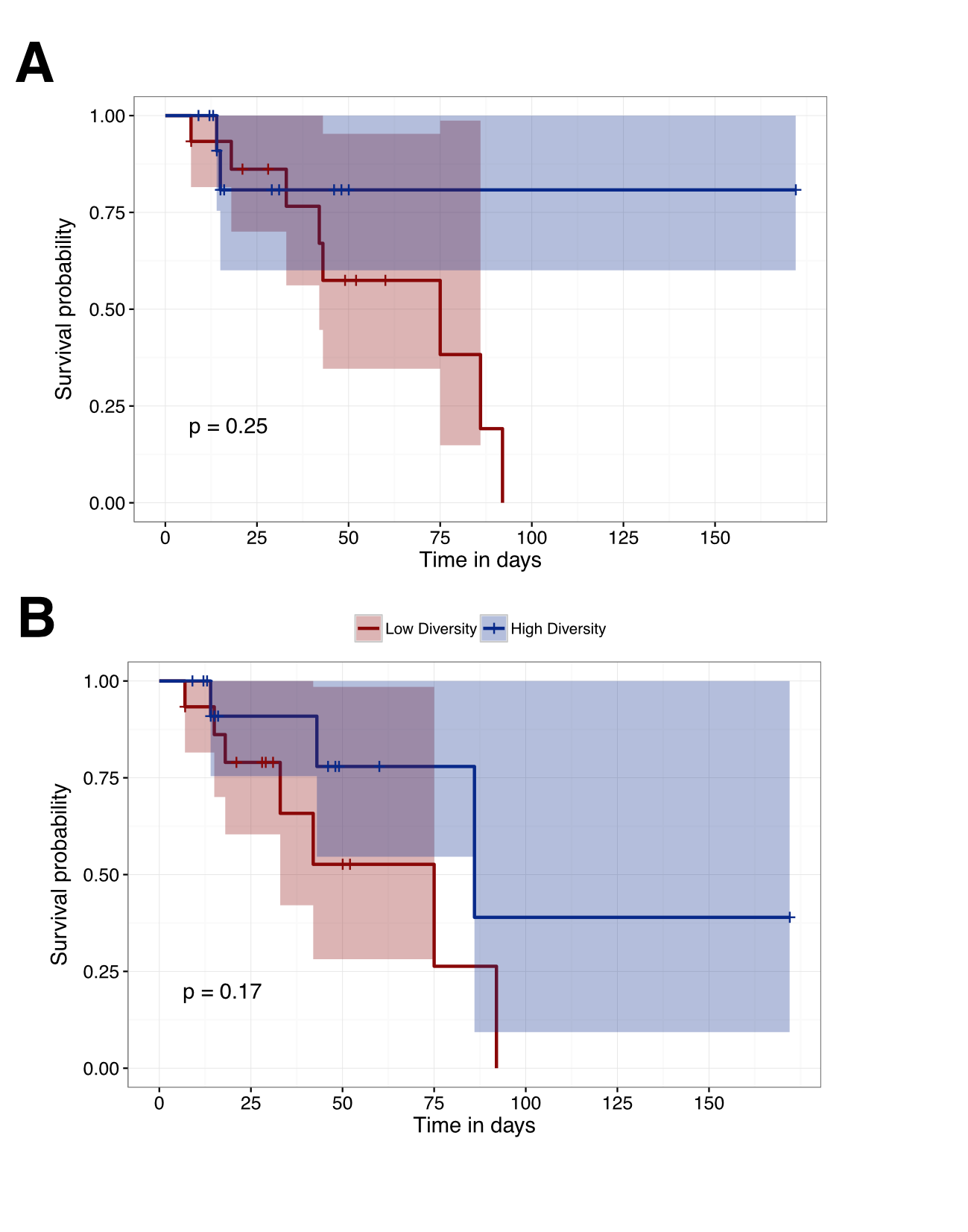


Figure S12
